# Simulation of Radiation-Induced DNA Damage and Protection by Histones Using the Code RITRACKS

**DOI:** 10.3390/biotech13020017

**Published:** 2024-06-05

**Authors:** Ianik Plante, Devany W. West, Jason Weeks, Viviana I. Risca

**Affiliations:** 1 KBR, 2400 NASA Parkway, Houston, TX 77058, USA; 2 Laboratory of Genome Architecture and Dynamics, The Rockefeller University, New York, NY 10065, USA; dwest@rockefeller.edu (D.W.W.); vrisca@rockefeller.edu (V.I.R.); 3 NASA Johnson Space Center, Houston, TX 77058, USA; jason.weeks-1@nasa.gov

**Keywords:** space radiation, DNA damage, single strand break, double strand break, direct effect, indirect effect, chromatin, epigenetics

## Abstract

(1) Background: DNA damage is of great importance in the understanding of the effects of ionizing radiation. Various types of DNA damage can result from exposure to ionizing radiation, with clustered types considered the most important for radiobiological effects. (2) Methods: The code RITRACKS (Relativistic Ion Tracks), a program that simulates stochastic radiation track structures, was used to simulate DNA damage by photons and ions spanning a broad range of linear energy transfer (LET) values. To perform these simulations, the transport code was modified to include cross sections for the interactions of ions or electrons with DNA and amino acids for ionizations, dissociative electron attachment, and elastic collisions. The radiochemistry simulations were performed using a step-by-step algorithm that follows the evolution of all particles in time, including reactions between radicals and DNA structures and amino acids. Furthermore, detailed DNA damage events, such as base pair positions, DNA fragment lengths, and fragment yields, were recorded. (3) Results: We report simulation results using photons and the ions ^1^H^+^, ^4^He^2+^, ^12^C^6+^, ^16^O^8+^, and ^56^Fe^26+^ at various energies, covering LET values from 0.3 to 164 keV/µm, and performed a comparison with other codes and experimental results. The results show evidence of DNA protection from damage at its points of contacts with histone proteins. (4) Conclusions: RITRACKS can provide a framework for studying DNA damage from a variety of ionizing radiation sources with detailed representations of DNA at the atomic scale, DNA-associated proteins, and resulting DNA damage events and statistics, enabling a broader range of future comparisons with experiments such as those based on DNA sequencing.

## 1. Introduction

DNA damage is an important consequence of exposure to ionizing radiation. Radiation induces the production of a variety of DNA lesions, notably single- and double-strand breaks (SSBs and DSBs) and various types of base damage [1]. DNA DSBs occur when two lesions are formed on opposite strands within 10 base pairs (bp), corresponding to one helical turn of the DNA [2]. Clustered DNA damage such as DSB+ and DSB++ occur when additional lesions are located no more than 25 bp away from the DSB [3]. It has been well established that DSBs are the most harmful type of damage to the cell [4,5]. Most DNA lesions, including DSBs, are repaired effectively by the cell. A small proportion of radiation-induced lesions are repaired very slowly [6,7]. Complex breaks that are difficult to repair are created in larger proportions by high-LET radiation [7,8]. Importantly, the mis-repaired lesions can be transmitted to daughter cells, which may lead to cancer [9].

The distinct spatial characteristics of the energy deposition, the track structure, from various types of radiations (high LET vs. low LET) have significant effects on the resulting damage to molecules [10,11], including DNA damage. When ionizing radiation (photon, electron, or heavy-ion particle) passes through matter, energy loss occurs in the form of ionizations and excitations [12]. Low-LET electromagnetic radiations such as γ-rays and X-rays ionize molecules largely via the Compton and photoelectric effects, which liberate electrons in the medium [13]. Low-LET radiations are relatively sparsely ionizing, creating local clusters of ionized molecules that have been called spurs, blobs, and short tracks in early track structure models [14]. High-LET charged particles, such as low-energy protons, α-particles, and ion-beams, produce a dense trail of ionizations that have a core of ionized and excited molecules and a surrounding penumbra of damage by secondary electrons [15]. The formation of track structure during the physical stage is very fast (~10^−15^ s). During the more or less overlapping physico-chemical stage, lasting ~10^−12^ s, radiolytic species such as the highly reactive hydroxyl (^●^OH) radicals are created. This is followed by the non-homogeneous chemical stage. In liquid water, often considered in radiation chemistry, this stage lasts ~10^−6^ s, corresponding to the time at which the radiolytic species are distributed homogeneously. However, in cells and biological media, the radicals have much more opportunities to react with biological molecules, so their lifetime is ~10^−9^ s [16].

Recently, there has been a renewed interest for radiation-induced DNA damage simulations [3,17,18,19,20,21]. An important aspect of the molecular context in which DNA damage occurs is how chromatin is organized with genomic DNA into 3-dimensional structures. Chromatin consists of DNA wrapped around histone proteins to form nucleosomes. Its structure comprises nucleosome positions, histones, and DNA modifications which constitute the epigenome of the cell and the three-dimensional organization of nucleosomes and chromatin fibers [22,23,24,25]. Differences in chromatin structure across the genome are responsible for cell-type-specific patterns of transcription and DNA repair [26,27,28,29]. Data from prior studies have also shown that chromatin structure influences the spatial pattern of DNA breaks caused by ionizing radiation [30,31,32,33,34]. Systematic epigenome mapping projects spanning multiple labs and institutions have produced data sets, making it possible to segment the genome-wide structural state of chromatin into discrete epigenetic states for a variety of human primary cells, cell lines, and tissues [22,29].

In this work, we present and discuss updates to the code RITRACKS (Relativistic Ion Tracks) [35] and apply it to simulate the DNA damage yield on nucleosomes with and without explicitly modeled histones. We refer to the updated code as RITRACKS version 4.0. (RITRACKS 4.0 is in the process of being released through the NASA software catalog). Several unique features of RITRACKS include the cross sections, the reaction rate constants, the transport algorithm, and the Green’s function approach for the radiation chemistry. Photons and ions (^1^H^+^, ^4^He^2+^, ^12^C^6+^, ^16^O^8+^, and ^56^Fe^26+^) were used for the simulation. The quantities of interest are the yield of damaged DNA structure (base, sugar, or phosphate); yield by cause of damage (ionizations, dissociative electron attachment (DEA), or chemical reactions) for DNA and amino acids; the SSB and DSB yields; and the locations of DNA breaks with respect to nucleosome structure and the DNA sequence. To assess whether DNA is protected from indirect radiation damage by the presence of histones through blocking of radical diffusion [17], the locations of the damage in base pairs were recorded. Comparison with other codes and experimental results were also performed. This shows that the framework can be used for subsequent simulation studies to model protein factors that modulate DNA damage propensity.

## 2. Materials and Methods

The code RITRACKS was used for this work. This code simulates detailed stochastic radiation track structure for photons, ions, and electrons, calculating the energy deposition events and the position of all radiolytic species generated [35] of all tracks in a pre-defined irradiated volume. The code also simulates radiation chemistry and micro- and nano-dosimetry. The modifications detailed below were made to simulate DNA damage at the atomic scale.

### 2.1. Simulation Setup

To calculate DNA damage yields values, an irradiation volume was defined, typically a parallelepiped with a surface of 0.5 µm × 0.5 µm and a length of 5 µm, in which 100 identical and isolated copies of the DNA fragments were placed randomly and with random orientations. The impinging particles (ions, electrons, or photons) entered from one end of the volume at random locations with trajectories parallel to the long axis of the volume. As typical yields values for DNA damage are in the order of ~10^−11^ breaks/Gy/bp, large DNA content and/or irradiation doses were necessary to limit statistical fluctuation of the results. Simulation of high doses and relatively large DNA content result in long calculation times. Therefore, the code RITRACKS was adapted to work on the NASA Langley cluster and on the NASA Space Radiation Analysis Group (SRAG) cluster, both being Linux parallel computer clusters. The number of central processing units (CPUs) can be determined by the user. For a typical calculation, 1000 CPUs are used for the calculation. Each CPU can also be assigned a number of simulation histories, each of which are independent simulations that can include multiple impinging particles. For this calculation, 10 histories per CPU were performed so that the number of histories was 10,000 in total. The typical dose used for the simulation is 10 Gy. The time required for a typical simulation to complete is from a few hours—for photons—to a few days for high-LET ions. The radiation chemistry simulation requires the largest amount of CPU time because a step-by-step method is used for the calculation.

### 2.2. DNA Models

For this work, only the B-form of DNA was considered, as it is the most common. RITRACKS has been updated with the capability to read atomic scale DNA and protein structures from files in the Protein Data Bank (PDB, www.rcsb.org) format, so it will be possible to use other DNA structures, proteins, or other molecular constituents of nucleoplasm in future projects. The 3D coordinates were read and used to calculate the center of each DNA structure and amino acid. Experimental metadata, such as the resolution, were also stored. In the program, the information of importance is the atomic location of DNA structures and amino acids. The DNA structure used in this work was the nucleosome structure 1kx5 (PDB https://doi.org/10.2210/pdb1KX5/pdb, accessed on 12 August 2021) [36].

### 2.3. Irradiation of a Volume

#### 2.3.1. Monte Carlo Simulation of Radiation Tracks

RITRACKS calculated the energy deposition events, ionization, and excitation of water molecules by protons and high-charge and energy (HZE) particles between 1 and 10,000 MeV/n, and for photons, between 0 and 1000 MeV. Additional calculations included the energies, positions, and directions of the secondary electrons, as well as the transport of the secondary electrons. Multiple ionizations were not included [37]. The code was previously validated with experimental data such as radial dose experiments [38]. Most calculations, such as the frequency of target hits and the mean energy deposited in the detector per event and per particle, were in good agreement with experimental data [39].

#### 2.3.2. Calculation of the Number of Tracks

The irradiation of the square surface of a parallelepiped volume of water by a collimated beam of ions was simulated as in recent work [40]. The number of ions impinging the bottom surface of the simulated volume for each Monte Carlo history was obtained by sampling the Poisson distribution:

(1) $$p\left(n\right)=\frac{\lambda^{n}e^{-\lambda }}{n!},$$

where λ=ϕA is the average number of tracks, ϕ is the fluence, and A is the area of the irradiated surface. For ions, the value of ϕ was obtained for a given dose D using the well-known equation,

(2) $$\phi \left({cm}^{-2}\right)=6.25\times 10^{8}D\left(Gy\right)/LET(keV/\mu m).$$

The LET was calculated from the Bethe equation, with corrections [41]. For photons, the number of initial photons for a simulation history was also obtained by sampling the Poisson distribution with λ=ϕA. The calculation of the initial fluence of photons is given in Appendix A.

#### 2.3.3. Periodic Boundary Conditions

The accurate dose calculation in micrometric volumes is of great importance for this work. Despite many improvements in the cross sections and parameters used in the code, the calculations made with RITRACKS without periodic boundary conditions (PBCs) underestimate the dose, notably for high-energy ions and for photons. This is because electrons that leave the irradiated volume can significantly lower the calculated dose. In radiation therapy or experiments, a large volume is usually irradiated, so there are electrons generated in neighbor volumes entering the volume of interest. To take this into account, PBCs have been implemented as an option of RITRACKS to obtain particle equilibrium. In summary, when an electron leaves the volume of interest, it re-enters the volume on the opposite side with the same energy and direction vector. It is possible for an electron to leave the volume many times; if this is the case, it will reenter as many times as necessary for all its energy to deposited in the simulated volume. A detailed discussion about PBCs and delta-electrons can be found in Plante et al. [40]. It should also be noted that PBCs are implemented for electrons that can originate from either photons or ions.

### 2.4. Overview of Radiation Interactions with Biomolecules

#### 2.4.1. DNA Damage by Direct Effect

Direct-type effects include both the direct effect, the direct ionization of DNA, and the quasi-direct effects, which include those ionizations of the first hydration shell and its near environment that transfer holes and non-solvated electrons to DNA [42]. Electron spin resonance (ESR) studies have suggested that the waters of hydration contributing to the quasi-direct effect extend out to 9 or 10 water molecules per nucleotide [43]. In this work, however, the direct effect refers to the direct interaction of the radiation track structure with the DNA, regardless of where the ionization occurs relative to the DNA.

#### 2.4.2. DNA Damage by Indirect Effect

The indirect effect of radiation primarily results from radical species produced in water radiolysis beyond 10 waters per nucleotide but still within a few nanometers of the DNA strand and can chemically react with the DNA. The radiation-produced hydroxyl radicals are the main damaging agent from the indirect effect [44,45]. To a lesser extent, electrons and hydrogen atoms formed by water ionization also contribute to the indirect effect [44,45]. As previously mentioned, in biological matter, the water radical species are largely scavenged by the high concentration of histone and other molecules in the DNA environment [42,44,45].

#### 2.4.3. Histone Damage

Histones are composed of amino acids and can be damaged by direct and indirect effects as is the case for DNA. In RITRACKS, amino acids are treated the same as DNA structures. Specifically, the cross sections and transport near amino acids and the radiation chemistry are performed in a similar manner. The details are given in Section 2.6.

#### 2.4.4. Histone Protection

Solvent accessibility of residues in globular proteins was introduced by Lee and Richards [46]. A molecule’s solvent accessible surface area (SASA) measures the contact area between molecule and solvent. There are many methods to calculate the SASA of a molecule, which are beyond the scope of this paper. The importance of SASA for this work is that DNA has a helical period of approximately 10 base pairs. Consequently, if a nucleosome is irradiated uniformly, the DNA bound to histones is not easily accessible for radical species, so a periodicity in the break patterns (as a function of the position of the base pairs) is expected. During the physical and physico-chemical stages, an electron can ionize an amino acid, reducing its energy without stopping it. Furthermore, another electron is generated during ionization. In contrast, DEA results in the electron binding to the molecule. The electrons remaining at 10^−12^ s become thermalized and hydrated. During the non-homogeneous chemical stage, radicals such as H^●^, ^●^OH, and e^−^_aq_ can react with the amino acids. These reactions may scavenge the radicals, preventing them from damaging DNA.

### 2.5. Simulation of DNA Damage

#### 2.5.1. Simulation of the Direct Effect

To simulate the interaction of the radiation track with DNA and histone proteins, the code RITRACKS was modified as described below. First, the cross sections of the interaction of the electrons and ions with the DNA bases and amino acids were included. Second, the transport algorithm was modified to simulate the trajectory of an electron or ion intersecting DNA and/or amino acids.

##### Ionization cross Sections

The ionization cross sections are based on the Binary-Encounter-Bethe (BEB) model [47]. In this model, the differential cross section dσ/dw is given as

(3) $$\frac{d\sigma }{dw}=\frac{s}{t+u+1}\left\{\frac{1}{{\left(t-w\right)}^{2}}+\frac{1}{{\left(1+w\right)}^{2}}-\frac{1}{1+t}\left[\frac{1}{t-w}+\frac{1}{1+w}\right]+\left[\frac{1}{{\left(t-w\right)}^{3}}+\frac{1}{{\left(1+w\right)}^{3}}\right]\right\}log(t).$$

In Equation (3), t, w, and u are the kinetic energy of the incident electron, the ejected electron, and the electron in the orbital, expressed in units of ionization potential (B) of the orbital. Hence, t=T/B, w=W/B, and u=U/B, where T, W, and U are the corresponding un-normalized quantities. Furthermore, $s=4\pi a_{0}^{2}NR^{2}/B^{2}$, $a_{0}=0.5292\;Å$ is the Bohr’s radius; R=13.61 eV is the Rydberg energy; and N is the number of electrons per molecular orbital, usually 2. The model parameters used in this work were taken from [48] and given in the Supplementary Materials.

The total ionization cross section for a molecular orbital was obtained by integration of Equation (3):

(4) $$\sigma =\int_{0}^{(t-1)/2}\frac{d\sigma }{dw}dw=\frac{s}{t+u+1}\left\{1-\frac{1}{t}+\frac{1}{2}\left[1-\frac{1}{t^{2}}\right]\log\left(t\right)-\frac{log(t)}{t+1}\right\}.$$

Since the incident and target electrons are indiscernible, the maximum energy was divided by two. Therefore, the integration limits were 0 and (t−1)/2.

An illustrative example of the differential and total ionization cross sections of thymine is provided in Supplemental Figure S1. Since a molecule comprises many electrons—each occupying molecular orbitals—the ionization cross section was calculated for each molecular orbital. For ionization to occur, the initial electron energy must have energy greater than the molecular electron’s potential energy. The total ionization cross section (TICS) was obtained by summing the ionization cross sections (Equation (4)) over all molecular orbitals. The total ionization cross sections for adenine, cytosine, guanine, thymine, and for the sugar and phosphate groups are shown in Supplemental Figure S2. They are similar in magnitude and shape to those reported in other work [49]. The TICS for amino acids are calculated in a similar way and included in Supplemental Figure S2.

When an ionization occurs, the energy of the secondary electron should be determined. At first, the molecular orbital being ionized is identified by building a table of cumulative probabilities for all orbitals at a given electron energy. The probabilities are given by the ratio of each molecular orbital to the total ionization cross section at that given energy. The sum of the probabilities is 1. Then, a uniformly distributed random number between 0 and 1 is generated and compared to the table of cumulative probabilities to determine which orbital is ionized. After determining the molecular orbital, the energy of the secondary electron is distributed as (dσ/dw)/σ. A value of w is generated using the sampling algorithm found in [47]. The total energy loss is for the initial electron is the sum of the ionization energy of the molecular orbital and the secondary electron energy.

Ions can also ionize DNA and amino acids. To obtain the cross sections for DNA and amino acid ionization by ions, the BEB equations were also used. The incident electrons’ energy was replaced by the energy of the ion per nucleon divided by the ratio of the mass between a proton and an electron. The rationale is that the cross section is dependent on the velocity of the incident particle. Furthermore, the cross section was multiplied by (Z*)^2^, given by

(5) $$Z^{*}=Z(1-exp\left(-125\beta^{2}Z^{-2/3}\right)),$$

where Z is the charge of the ion, and *β* is the ratio of the velocity of the ion to the speed of light [50]. The scaling factor $Z^{*}$ was used instead of Z to consider the charge screening resulting from electron capture from the medium by the ion at low energies. However, the charge screening effect is not very important at the energies used in this work, so $Z^{*}$ is essentially equal to Z.

##### Dissociative Electron Attachment cross Section

Many electrons, notably in electron track ends and secondary electrons from ionization of water and DNA molecules, do not have sufficient energy to further ionize molecules. These low-energy electrons can interact with the DNA structure by other mechanisms, such as DEA and elastic collisions (discussed in the next sub-section).

In their landmark paper, Boudaïffa et al. [51] showed that low-energy electrons (≤20 eV) can damage DNA by DEA, a topic which is subject to extensive theoretical and experimental work. DEA cross sections were implemented in RITRACKS for DNA bases, sugar, phosphate, and the amino acids. It is noteworthy that the DEA cross sections are several orders of magnitude lower than ionizations or elastic cross sections.

**Figure 1:**
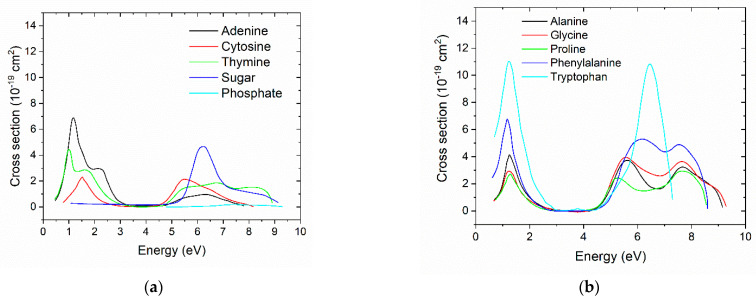
(**a**) DEA cross sections for adenine, cytosine, thymine, sugar, and phosphate from [52]. (**b**) DEA cross sections for alanine, glycine, proline, phenylalanine, and tryptophan from [53]. The graphics are on the same scale for comparison.

For DNA, the DEA cross sections from Aflatooni et al. [52] were used. For thymine, adenine, and cytosine, the cross sections were extracted from the graphics in this paper. The cross section for guanine was not provided; therefore, the DEA cross section of adenine was used. For sugar, we used the 3-hydroxyTHF cross section; for phosphate, we used the trimethylphosphate cross section. The DEA cross sections are known for selected amino acids (tryptophan, phenylamine, proline, alanine, and glycine) [53]. For the other amino acids, since the cross sections are not known, the cross sections of amino acids with similar molecular structure were used. The DEA cross sections are shown in Figure 1, and details are included in the Supplementary Materials. Because the DEA cross sections were much smaller than the corresponding ionization and elastic ones, we did not expect the exact shape to influence the results. However, the DEA cross sections reported by Liu et al. (2017) [54] were several orders of magnitude higher than those used in this work. The cross sections reported by Aflatooni et al. [52] and Sheer et al. [53] were measured in the gas phase, whereas those in Liu et al. used collected values from several groups on different studies, including the gas phase and condensed phase.

##### Elastic cross Sections

**Figure 2:**
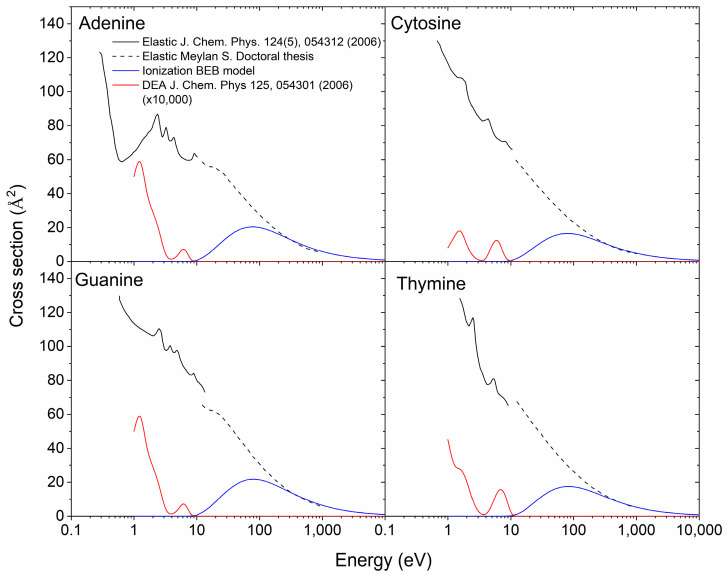
Ionization [47], elastic [56,57], and DEA [52] cross sections for adenine, cytosine, guanine, and cytosine. The DEA cross sections are considerably lower than the ionizations and elastic cross sections and have been multiplied by 10,000.

The elastic cross sections for DNA bases were calculated by the phase-shift method in Możejko and Sanche [55]. Although some numerical values are given in this paper, this calculation is complicated to use in RITRACKS because it involves solving a differential equation numerically. Furthermore, these cross sections calculated by the phase-shift method are not likely to be valid at low energies. The elastic cross sections used for this work were taken from two different sources. At low energies (<10 eV), the cross sections for adenine, cytosine, guanine, and thymine are taken from Tonzani and Greene [56]; at higher energies (>10 eV), they were taken from Meylan [57] (Figure 2). The elastic cross sections from the two different sources were similar at the junction point around 10 eV. The elastic cross sections for sugar and phosphate were also taken from Meylan [57]. However, they covered only the higher energy range. At energies below the lowest energy for which the cross sections are known—less than 1 eV—the cross sections were taken as a constant equal to the known value of the cross section at the lowest energy. For amino acids, the elastic cross sections were not found. Based on available data [57], the elastic cross sections are roughly proportional to the molecular weight; therefore, the cross sections for amino acids were taken using the thymine cross section, multiplied by the ratio of the molecular weight of the amino acid and thymine.

##### Particle Transport in Media of Different cross Sections

**Figure 3:**
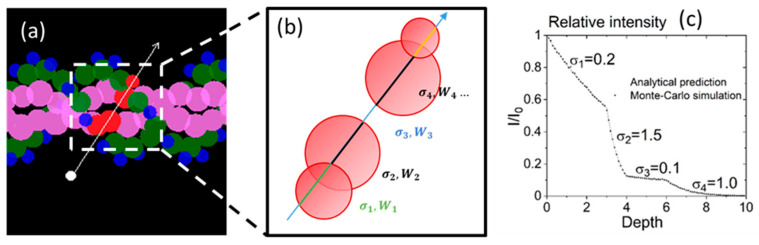
(**a**) An electron’s trajectory intersects a sugar (green), two bases (pink), and one phosphate group (blue). (**b**) The sequence of media seen by the electron, of total cross section ($\sigma_{i}$) and width ($W_{i}$). The colors of the trajectory indicate that the cross section is different. (**c**) Sampling of distance in a medium comprising four different cross sections. The cross sections $\sigma_{1}\ldots \sigma_{4}$ are given here in arbitrary units. The line is given by Equation (8). The dots are the distances from the original position as determined by the sampling algorithm.

To simulate the interaction of the radiation track with DNA, the code RITRACKS was modified. The DNA structure is represented as spheres for the bases, sugar, and phosphates (Figure 3). During the particle transport simulations, the code determines the DNA structures intercepted by an electron or ion trajectory.

When the DNA structure was intercepted by the electron or ion trajectory, the intersection points of the trajectory and the spheres were computed, and a list of the media traversed by the electron trajectory was built with their total cross section ($\sigma_{i}$) and width ($W_{i}$) (Figure 3).

To simulate particle transport in this non-homogenous media, we consider a beam of particles of intensity I. The number of particles in the beam is reduced when the particles interact. This is described by the usual differential equation for particle transport:

(6) $$dI=-\sigma \left(x\right)NIdx,$$

where N is the density of targets, x is the distance from the original position of the particle, and $\sigma \left(x\right)$ is the total cross section. For a constant cross section, solving Equation (6) leads to an exponential decay. For n different media traversed by the particle (excluding the last one, which is the original medium of infinite width),

(7) $$\sigma \left(x\right)=\left\{\begin{array}{cc} \sigma_{0} & 0\leq x<W_{1}\\ \begin{array}{c} \sigma_{1}\\ \sigma_{i}\\ \sigma_{0} \end{array} & \begin{array}{c} W_{1}\leq x<{W_{1}+W}_{2}\\ \sum_{j=1}^{i}W_{j}\leq x<\sum_{j=1}^{i+1}W_{j}\\ x\geq \sum_{j=1}^{n}W_{j} \end{array} \end{array}\right..$$

The cross section $\sigma_{0}$ is for the medium in which the DNA is located (water for the simulations). As the trajectory ends in the original medium, the width of the last media is infinite. The trajectory usually starts in the original media, but it is not necessarily the case following an interaction within a DNA structure. In that case, the initial $\sigma_{0}$ is replaced by the appropriate cross section. The integration of Equation (6) using $\sigma \left(x\right)$ given by Equation (7) is straightforward and yields exponentials:

(8) $$I(x)=I_{0}exp\left[-\sum_{j=1}^{i}\left(\sigma_{j}-\sigma_{i}\right)NW_{i}-\sigma_{i}Nx\right],$$

where i is the index of the medium in which x is found.

Using the sequence of widths and cross sections, RITRACKS samples the position where the next interaction will take place using the algorithm described previously [47]. Briefly, the relative weight $p_{i}$ of each zone, which determines where the interaction will happen, is given by:

(9) $$p_{i}=I_{0}\frac{exp\left[-N\sum_{j=1}^{i}\left({W_{j}\sigma }_{j}\right)\right]\left[1-\exp(-NW\sigma_{i+1})\right]}{N\sigma_{i}}.$$

Once the zone of the interaction (*i*) is determined, the exact position ($W_{s}$) is found by:

(10) $$W_{s}=\sum_{j=1}^{i-1}W_{j}-\frac{1}{N\sigma_{i}}log\left[1-V(1-\exp(-N\sigma_{j}W_{j}))\right],$$

where *V* is a random number uniformly distributed between 0 and 1. An example of distance sampling results is shown in Figure 3 for validation of the algorithm.

In contrast, in the work using Geant4-DNA [19,58], a direct strand break was formed if at least 17.5 eV of deposited energy was accumulated within the backbone volume and neighboring hydration shell. Nikjoo et al. [59] also assumed that a break occurs when the energy deposited by a track stochastically in one sugar-phosphate volume exceeds a threshold value of 17.5 eV. Similarly, an early version of the code PARTRAC used an energy deposition threshold of 9.5 eV [60]. An approach, used in a newer version of PARTRAC [61] and in Geant4-DNA [62], used a linearly increasing probability distribution, where there was a 0% probability a break occurred when less than 5 eV was deposited, and a 100% probability a break occurred when over 37.5 eV was deposited in the sugar phosphate moiety. Also, the current public version of Geant4-DNA named “molecularDNA” does not yet include the DNA cross sections [63].

#### 2.5.2. Simulation of the Indirect Effect

To simulate chemical reactions between the radiolytic species and DNA bases, we use an approach based on the Green’s functions of the diffusion equation (GFDE), in which distance and time-dependent probability of reactions are used instead of diffusion approaches purely based on reaction radius commonly used in simulation of water radiolysis [64]. The GFDE approach is generally considered to be the gold standard to validate other theories used in chemical reaction simulations [65,66,67] and has been used to simulate biochemical networks in time and space [68].

The Monte-Carlo sampling algorithm for the GFDE of reversible reactions with an intermediate state [69] was adapted to simulate the radiation chemistry as well as DNA damage by free radicals. This is done by using the known reaction rate constants between radicals (^●^OH, e^−^_aq_, H^●^, …) and the DNA bases, sugars, and phosphates, given in the Supplementary Materials. The sampling algorithms used to simulate the diffusion of free radicals and chemical reactions with DNA are detailed in earlier work [70,71]. For the reactions between the radicals and the DNA, which are partially diffusion-controlled, the Green’s function is given by [65]

(11) $$4\pi rr_{0}p(r,t|r_{0})=\frac{1}{\sqrt{4\pi Dt}}\left\{exp\left[\frac{{\left(r-r_{0}\right)}^{2}}{4Dt}\right]+exp\left[\frac{{\left(r+r_{0}-2R\right)}^{2}}{4Dt}\right]\right\}+\alpha W\left(\frac{r+r_{0}-2R}{\sqrt{4Dt}},-\alpha \sqrt{Dt}\right),$$

where $\alpha =-(k+4\pi RD)(4\pi R^{2}D)$, k, R, and D are the reaction rate constant, reaction radius, and diffusion coefficient. The functions $Erfc\left(x\right)$ and $W\left(x,y\right)$ are defined as:

(12a) $$W\left(x,y\right)=\exp\left(2xy+y^{2}\right)Erfc\left(x+y\right),$$

(12b) $$Erfc\left(x\right)=\frac{2}{\sqrt{\pi }}\int_{x}^{\infty }e^{-u^{2}}du.$$

The quantities r and $r_{0}$ are the final and initial distance between one particle and another or a DNA structure. Hence, $p\left(r,t|r_{0}\right)$ is the probability that a particle will be found at distance r from another particle or a DNA structure at time t, given that they were initially separated by the distance $r_{0}$. A sampling algorithm for r is given in [71]. If many particles are involved, an average position weighted by the initial distance is calculated. The probability of reaction at each time step is obtained by integration:

(13) $$P\left(r_{0},t\right)=1-\int_{R}^{\infty }4\pi r^{2}p\left(r,t|r_{0}\right)dr=\frac{R\alpha +1}{r_{0}\alpha }\left[W\left(\frac{r_{0}-2R}{\sqrt{4Dt}},\alpha \sqrt{Dt}\right)-Erfc\left(\frac{r-r_{0}}{\sqrt{4Dt}}\right)\right].$$

In general, the simulations should be independent of the number of timesteps used. The time discretization properties are discussed in previous research [72]. For a pair of particles that can react according to the GFDE approach, the following equations hold:

(14a) $$p\left(r_{2},\Delta t_{1}+\Delta t_{2}|r_{0}\right)=\int_{R}^{\infty }4\pi r_{1}^{2}p\left(r_{2},\Delta t_{2}|r_{1}\right)p\left(r_{1},\Delta t_{1}|r_{0}\right)dr_{1},$$

(14b) $$P\left(r_{0},\Delta t_{1}+\Delta t_{2}\right)=P\left(r_{0},\Delta t_{1}\right)+\int_{R}^{\infty }4\pi r_{1}^{2}P\left(r_{1},\Delta t_{2}\right)p\left(r_{1},\Delta t_{1}|r_{0}\right)dr_{1},$$

That is, Equation (14) expresses that a pair with initial separation $r_{0}$ can either

(1) Go to $r_{1}$ during $\Delta t_{1}$ and then $r_{2}$ during $\Delta t_{2}$; or
(2) React during $\Delta t_{1}$ or go to $r_{1}$ during $\Delta t_{1}$ and then react during $\Delta t_{2}$.

In contrast, in the work of Zhu et al., [58], using Geant4-DNA, only the interactions between the ^●^OH radicals and the DNA backbone were assumed to induce indirect strand breaks. Furthermore, the probability for a reaction with the DNA to induce a strand break is 0.4 [62,73]. Earlier work by Nikjoo et al. [59] used a probability of 0.13. Also, in the work of Sakata et al. [17], using Geant4-DNA, all the radicals entering the histone region were terminated.

In typical radiation chemistry simulations, the calculations are usually performed until 10^−6^ s, the time at which most spur reactions are complete [74]. For cellular DNA damage simulations, radicals are unlikely to live that long because they react with other biomolecules. Therefore, the simulations were limited to 10^−9^ s in this work, as in Nikjoo et al. (2001) [59] and Zhu et al. (2020) [58]. In comparison, other groups have simulated non-homogenous chemistry up to 2.5 × 10^−9^ s [18,62] and 5.0 × 10^−9^ s [17] in DNA damage simulations. Several techniques may be used to reduce the number of timesteps in a simulation [75] with the aim of obtaining sufficiently precise results while keeping the calculation time reasonable. For this work, 100 timesteps per decade from 10^−16^ to 10^−9^ s (total 700 timesteps) were used. Sakata et al. (2020) [17] have also used the independent reaction times (IRT) method for the chemistry simulation, which is much faster than the step-by-step (SBS) method. Although the IRT method is also implemented in RITRACKS, it was not used in this work.

#### 2.5.3. Classification of DNA Damage

DNA damage is frequently classified based on the proximity of DNA lesions, regardless of origin. For this work, we applied the classification used by Nikjoo [1]. Briefly, a single-strand break (SSB) was essentially an isolated lesion (resulting from an ionization or reaction) in the DNA. Two lesions were considered SSB+ when they were on the same strand. When strand breaks were located on different strands and were 10 bases pairs apart or less, they were considered double-strand breaks (DSB). When they were separated by more than 10 base pairs, they were considered 2SSB. More complex breaks, such as DSBs with an additional lesion (DSB+) and two additional lesions (DSB++) on opposite strand and within 10 bp, were also calculated, but they are not reported in this work.

### 2.6. Simulation of Histone Damage

An important part of this work was to assess the possible protection of DNA by the histone proteins, which are composed of amino acids. Essentially, the approach used for DNA bases was also used for all amino acids. The amino acids used in this work were alanine, arginine, asparagine, aspartic acid, cysteine, glutamic acid, glutamine, glycine, histidine, isoleucine, leucine, lysine, methionine, phenylalanine, proline, serine, threonine, tryptophan, tyrosine, and valine. For the chemistry, the reaction rate constants between H^●^, ^●^OH, and e^−^_aq_ with every amino acid are known. Therefore, all possible reactions between these radicals and the amino acids have been included. The parameters are given in the Supplementary Materials. In contrast, Sakata et al. (2020) [17] performed simulations using Geant4-DNA considering the histones as perfect scavengers for all radiolytic species, meaning that, in the simulations, any free radiolysis species that entered a histone region (modelled as a 2.5 nm radius sphere) was stopped and terminated.

### 2.7. Fragment Length Distribution

The fragment length of each strand of each DNA molecule was recorded, assuming that a lesion led to a break in that specific DNA strand. Furthermore, the DNA strands with a break at both extremities were recorded in another distribution. The latter was used for comparison with the Radiation-Induced Correlated Cleavage with sequencing (RICC-seq) [30] experiment results. Briefly, RICC-seq is a protocol for sequencing and mapping the single-stranded DNA fragments flanked by single-strand breaks induced by irradiation of mammalian cells with ionizing radiation [30]. For the data used here, human BJ fibroblasts were irradiated with 300 Gy of 0.66 MeV photons from a ^137^Cs source while chilled on ice and then were immediately lysed to extract DNA. Genomic DNA was protected by embedding the cells in agarose plugs. The DNA was briefly denatured at 95 °C to release single-stranded DNA fragments, which were concentrated, ligated to double-ended sequencing adapters using splint ligation, sequenced using Illumina short-read paired-end sequencing, and mapped to the human genome reference (hg19).

### 2.8. Damage Location

The damage location was recorded by base pair number along the DNA, for each strand, and compiled for all histories in a simulation. To assess periodicity in the break patterns, the Discrete Fourier Transform (DFT) of the number of lesions as a function of the base location was calculated for each strand.

### 2.9. Error Bars

For all the results presented in this work, the error bars were calculated as the standard error of the mean multiplied by 1.96. This yielded a 95% confidence interval on the calculated values.

## 3. Results

In this section, the simulation results calculated by RITRACKS 4.0 are presented. The 100 copies of the nucleosome structure 1kx5.pdb (PDB https://doi.org/10.2210/pdb1KX5/pdb, accessed on 12 August 2021) [36], which comprises 147 bp, were placed in a volume encompassing the DNA structure and irradiated by photons or ions. The ions selected for the irradiation were ^1^H^+^, ^4^He^2+^, ^12^C^6+^, ^16^O^8+^, and ^56^Fe^26+^.

### 3.1. Damage by Structure Type and by Contribution

**Figure 4:**
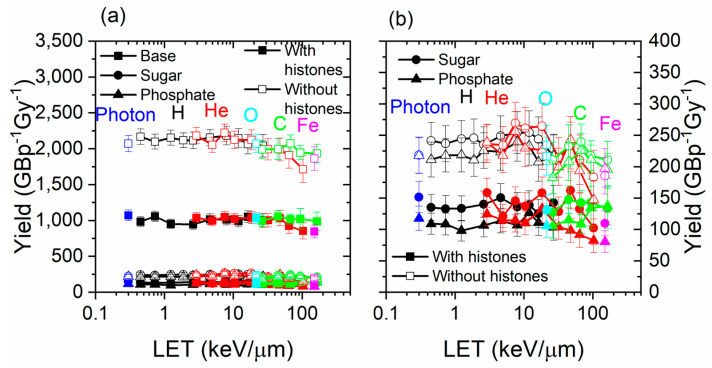
(**a**) Yield by DNA structure damaged (base, sugar, or phosphate) for photons, ^1^H^+^, ^4^He^2+^, ^12^C^6+^, ^16^O^8+^, and ^56^Fe^26+^ as a function of the LET, with and without histones. (**b**) The figure on the right is a smaller scale version of the figure on the left and focuses on sugar and phosphate.

Damage to DNA by structure type (base, sugar, or phosphate) is shown in Figure 4. The damage recorded includes direct and indirect effects, as well as DEA. In general, the damage yield by type of structure damaged did not greatly vary with the LET. The damage yield was much higher for the bases than for sugar and phosphates. This was expected since the sugar and phosphates were smaller than the bases, so it was more difficult for an electron track to intercept it or for a chemical reaction to occur. The yield of damage was much higher (approximately double) for all structure types when histones were not present.

**Figure 5:**
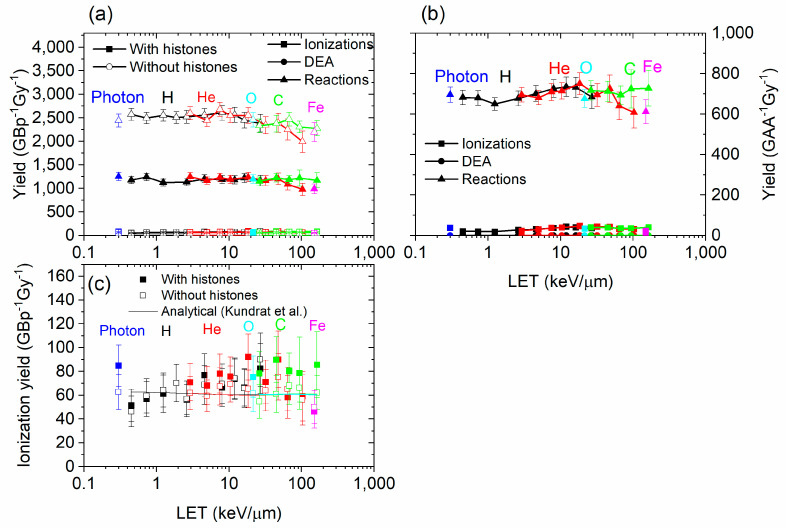
Yield by cause of damage (ionizations, DEA, or chemical reactions) for DNA (**a**) and for amino acids (**b**), for ^1^H^+^, ^4^He^2+^, ^12^C^6+^, ^16^O^8+^, and ^56^Fe^26+^, as a function of the LET, with and without histones. (**c**) is identical to (**a**), at a different scale, and includes analytical calculations from Kundrát et al. [76].

The contributions of causes of DNA and amino acid damage are shown in Figure 5. The contribution by cause did not vary much with the range of LET studied. The simulation results from Geant4-DNA and PARTRAC showed a similar trend with the LET [17,61]. However, simulation results obtained with TOPAS-nBio, which is based on Geant4-DNA, showed a peak in the breaks obtained by indirect effect at a LET value of 5 keV/µm [58]. Chemical reactions are the most important type of damage to the DNA and amino acids. Without histones, the yield of damage to DNA did not greatly change for DEA and ionizations, but it increased by a large factor for reactions. The yield of reaction damage increased from ~1200 to ~2500 GBp^−1^Gy^−1^ when histones were not present. Therefore, the histones prevented about half of the DNA damage by reactions with chemical species generated by irradiation. This suggests that the direct effect is minimally impacted by the presence of histones, while the indirect effect is strongly impacted. This was expected since reactions with amino acids result in the absorption of the radicals, preventing them from interacting with DNA. However, the consequence of the presence of histones on the direct effect is more difficult to assess. As the ionization cross sections of the amino acids were higher than water, more ionizations occurred when histones were present than when they are absent, subsequently creating more electrons; however, most of these ionized electrons had low energy, and many of them could not further ionize the DNA. They could perform a DEA with other amino acids or the DNA structure, although the DEA cross sections were very low, and elastic interactions—which did not lead to damage—were much more likely.

### 3.2. Damage Types

**Figure 6:**
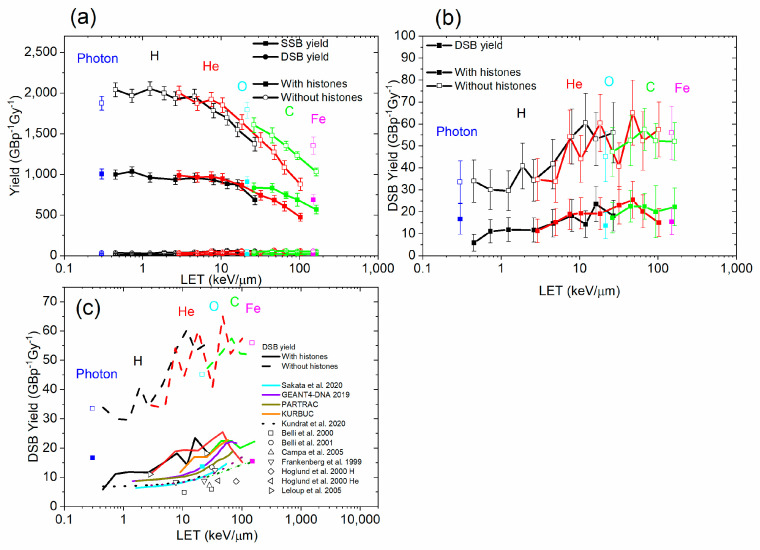
(**a**) SSB and DSB yield, for photons, ^1^H^+^, ^4^He^2+^, ^12^C^6+^, ^16^O^8+^, and ^56^Fe^26+^, as a function of the LET, with and without histones. (**b**) DSB yields for photons and the same ions as a function of the LET, with and without histones. (**c**) Comparison of RITRACKS 4.0 DSB yield with calculations from other codes [17,59,61,75] and experiments [79,80,81,82,83,84].

The yields of damage by type (SSB, DSB) as a function of the LET are shown in Figure 6. In general, the SSB yields decreased, and the DSB yields increased with the LET of the radiation. This indicates that the complexity of breaks tends to increase with the LET of radiation. That is likely due to the proximity of the breaks at higher LET. Both SSB and DSB yields were higher in the simulations without histones. The DSB yield calculated by RITRACKS in the absence of histones was higher than most experimental data and calculations by other codes. However, values of 30–60 DSBs/Gy/Cell, corresponding to 5–10 DSBs/Gy/GBP, were reported previously by Margis et al. [77] for low-energy electrons. The results were also very noisy because DSBs require two breaks, requiring a large number of simulation histories. Most results for DSBs obtained with RITRACKS were higher than corresponding experimental data and simulation results from other codes. This could be due to several factors. The results are dependent on the radiation chemistry parameters, such as the timestep and final simulation time, and the approach used in other codes. Other factors could include how the normalization is performed to obtain the yields. Experimentally, other factors intervene. Notably, the DNA lesions do not necessarily remain when they have been created. For examples, holes produced during the initial ionizing event in DNA, for the most part, transfer to the base with the lowest ionization potential, guanine [78]. The added electrons (for example, in DEA events), usually transfer to the bases of highest electron affinities, i.e., the pyrimidines, thymine, and cytosine. Losses of DNA fragments in sample processing can also be challenging to estimate experimentally, and it is therefore possible that RITRACKS is predicting some breaks producing fragments that would become lost in processing.

### 3.3. Histone Protection

**Figure 7:**
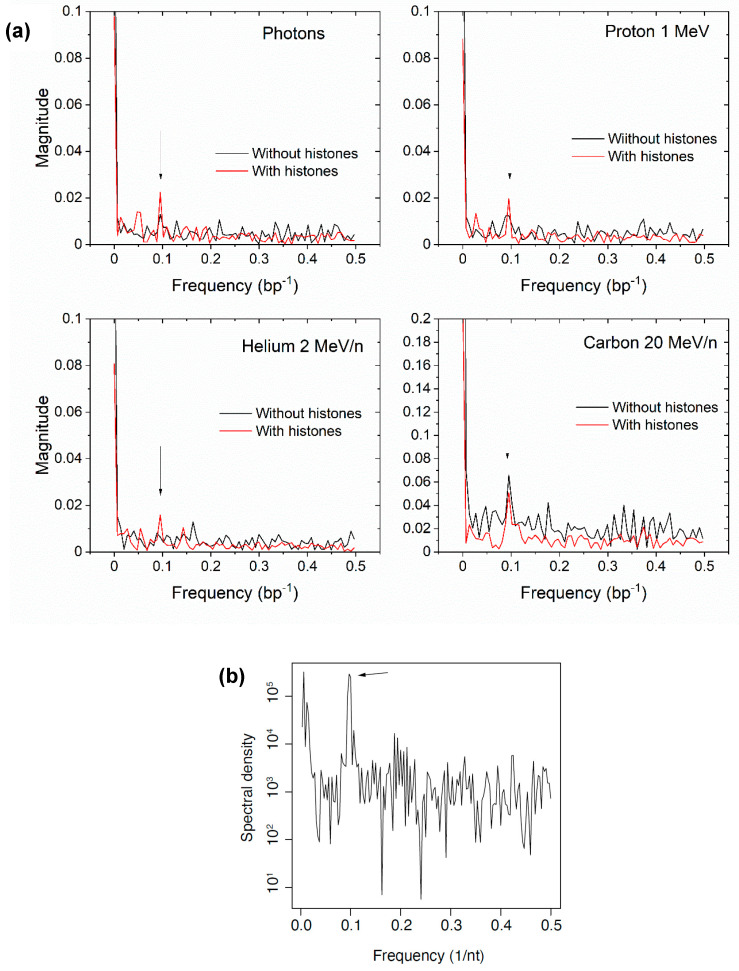
(**a**) DFT of the damage base pair number for 100 keV photons, ^1^H^+^, ^4^He^2+^, and ^12^C^6+^. The peak at 0.1 bp^–1^ indicate periodicity of 10 bp, suggesting histone protection. (**b**) DFT (periodogram) of single-strand DNA fragment ends positions aligned with respect to nucleosomes mapped using ATAC-seq [30]. The fragments were captured from intact human BJ fibroblast cells irradiated with 300 Gy 0.66 MeV γ-ray photons from a ^137^Cs source while on ice and immediately lysed, prepared for DNA sequencing with a RICC-seq protocol [30]. A linear scale is used for the simulation results in to put the peak in evidence.

Taken together, the results shown in Figure 4, Figure 5 and Figure 6 display the effects of histone protection of DNA on the overall yield of DNA damage events. We then sought to investigate the spatial distribution of breaks along the DNA sequence as well as in three dimensions around the nucleosome. In a nucleosome, the DNA in contact with the histones are more protected than those facing the outside of the nucleosome. Therefore, because the DNA is a helix with a period of approximately 10 bp, a periodic pattern in breaks as a function of the base pair number was expected. To assess the periodicity, we took the discrete Fourier transform (DFT) of the damage vs base pair number for photons and selected ions. The results are shown in Figure 7. Although the results were noisy, in most simulations, a peak was observed at a frequency of 0.1, suggesting that the DNA damage occurred at periodic locations along the DNA, with a frequency matching the helical turn of the DNA. When the simulation was performed without histones, the peak was less prominent relative to background noise. This result supports the model of local, base-pair-specific histone protection of DNA.

**Figure 8:**
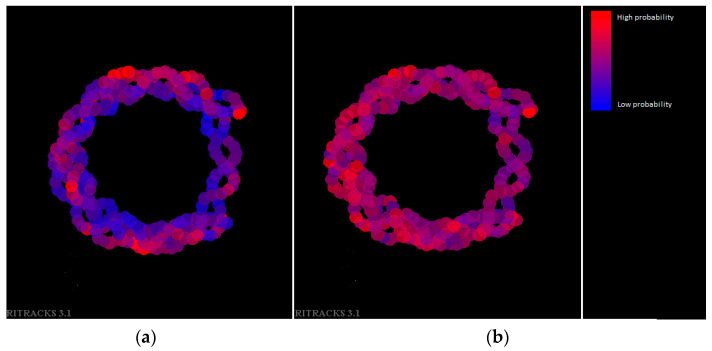
Nucleosomal DNA, with color function of the probability of having damage. Blue indicates a low probability of damage (including 0), and red indicates a high probability of damage (up to the maximum value for this simulation, 0.007). (**a**) Simulation with histones present. (**b**) Simulation without histones present. The ion used is ^12^C^6+^, 50 MeV/n.

We also plotted the DNA wrapped around a nucleosome with a color map representative of the probability of a break (Figure 8). The ion used was ^12^C^6+^, 50 MeV/n. In general, when histones were absent, all DNA had a roughly equal probability to be damaged. When histones were present, the DNA in the periphery had a much higher probability of damage than those located in the center or near the histone proteins. Although Figure 8 broadly shows the contrast of damage probabilities between the inner and outer parts of the nucleosome, the precise amount of damage was subject to statistical fluctuations and could also be dependent on factors such as the specific DNA sequence and the amino acids that are in proximity of the DNA. The variation in damage density around the nucleosome within each condition (with or without histones) was likely due to statistical fluctuations but could have also been due to partial protection by the histone proteins extending beyond the core of the nucleosome. This simulation used a single static structure of the nucleosome core (PDB 1KX5), but in physiological conditions, fluctuations of the unstructured domains of histones would likely average out such local protection.

### 3.4. Fragment Length Distribution

**Figure 9:**
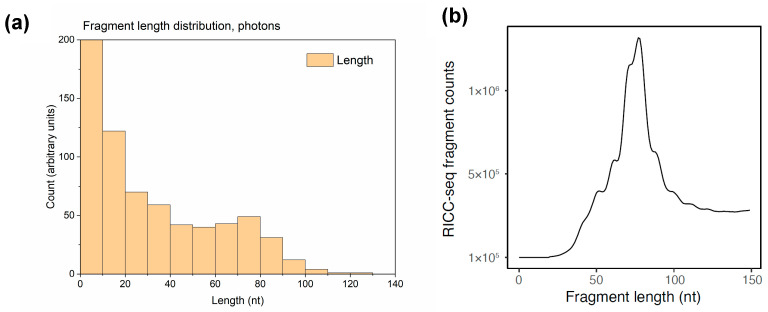
(**a**) Calculated single-stranded DNA (ssDNA) fragment length distribution from single nucleosomes in RITRACKS 4.0 simulations with 100 keV photons. (**b**) Experimental fragment length distribution from human BJ fibroblasts irradiated with 300 Gy of 0.66 MeV γ-ray photons from a ^137^Cs source while kept on ice, immediately lysed, and processed with the RICC-seq protocol to sequence the resulting ssDNA fragments [30]. Fragments below ~40 nt were size-selected away during sequencing library preparation and were not detected. The peak at ~78 nt corresponded to approximately one turn of DNA around the nucleosome core.

In experiments that used either electrophoresis [31] or DNA sequencing (RICC-seq) [30] to determine the length distribution of single-strand DNA (ssDNA) fragments resulting from irradiated human cells, several peaks were observed. The largest one was located at about 78 nucleotides (nt) and corresponded to one helical turn of the DNA around the histone. Therefore, this indicates that correlated DNA damage events that occurred at proximal loci on the first and second gyre of each strand of DNA wrapped around the nucleosome. To investigate whether we could observe this peak in the fragment length distribution, ssDNA fragment length calculation was added in the DNA damage analysis section of RITRACKS. An example of such calculation for the nucleosome structure 1kx5.pdb is shown in Figure 9.

The calculated fragment length distribution showed a peak at about 78 nt, as seen in the experiments. Because of computing limitations, the peak in the fragment length distributions was more difficult to see in the simulation than in the experiment due to lower statistics. For this calculation, the DNA was composed by one nucleosome, so the maximum fragment size was 140 nt. Therefore, other peaks beyond 140 nt were not seen. Furthermore, the experimental data are missing the large number of fragments below ~40 nt because the RICC-seq protocol involves size-selecting out the DNA molecules below that length while the sequencing library is constructed [30]. Similar fragment length distribution patterns were observed in simulations with other ions and energies.

## 4. Discussion

This work presents the capabilities of RITRACKS 4.0 to simulate radiation-induced DNA damage. As more thoroughly described in the Supplementary Materials, we have updated the code with several features, including the ionization energies for each molecular orbitals of each type of molecule and the reaction rate constants with radicals, to explicitly account for the complex interactions of ions and photons with DNA and protein.

Due to the complexity of the simulations and the large number of parameters, it was nearly impossible to individually study the effect of these parameters on the results because a sensitivity analysis would have to be performed for each individual parameter to be tested. This would require extensive simulations that are currently unfeasible. We have therefore taken advantage of the fact that these parameters are known or estimated for individual DNA structures and amino acids. One limitation of the study is that the parameters may change somewhat when the nucleotides or amino acids are part of a macromolecule such as a DNA fragment or chromatin fiber; however, the current estimates are the best available and represent advancement beyond prior versions of the code that did not explicitly account for the cross-sections and interaction types described in this paper. Several features included in RITRACKS, such as the cross sections for amino acids, the reactions between radicals and the amino acids, and the step-by-step GFDE method for radiation chemistry, to our knowledge, have not been implemented in other codes.

Many other factors in the simulation setup, such as the irradiation volume, the type and energies of the radiation, and the orientation of the DNA fragments, were expected to influence the results. Exhaustive exploration of these parameter effects was not feasible due to the computationally intensive nature of these simulations. The damage yield was ~10^−3^/nucleosome/Gy. This means that for a dose of 1 Gy, about 1000 nucleosomes should be irradiated to record one damage. The dose can be increased artificially using very small irradiation volumes. In the simulations for this work, the doses were in the order of 10 Gy, but volumes that are too small necessarily lead to boundary effects. Also, radiation chemistry simulations are notoriously slow because the number of pairs in a system of N particles is N(N − 1)/2, i.e., roughly proportional to N^2^. With 10,000 histories, we were able to achieve good statistics for SSBs and acceptable convergence for DSB error bars to clearly distinguish conditions.

For the chemistry, the number and magnitude of timesteps are also important. We performed a study of damage yields as a function of the number of timesteps (included in the supplemental document). We observed that the number of timesteps had a small influence on the damage yields and that there was no advantage of using more than 100 timesteps per decade.

Although we have significantly expanded the types of interactions and reactions modeled in RITRACKS, it is important to consider that the damage to a DNA structure does not necessary stay where it has been created. For examples, holes produced during the initial ionizing event in DNA, for the most part, transfer to the base with the lowest ionization potential, guanine [78]. The added electrons (for example, in DEA events) usually transfer to the bases of highest electron affinities, i.e., the pyrimidines, thymine, and cytosine. As this is not currently included in the model, some DNA sequence biases observed experimentally may not be seen in the simulation. Such details will need to be addressed in future work.

The simulation results show that, in general, the yield of damage by chemical species did not change much with LET. The simulation results from Geant4-DNA and PARTRAC showed a similar trend with the LET [17]. However, simulation results obtained with TOPAS-nBio, which is based on Geant4-DNA, show a peak in the breaks obtained by indirect effect at a LET value of 5 keV/µm [58]. The chemistry part is probably dependent on the simulation parameters such as the final simulation time and the timestep. Moreover, the yield of damage by cause (ionization or chemical reaction) did not vary much with the LET. All yield values decreased when histones were included in the simulation. The yield decreased from ~2500 to 1200 damage/Gbp/Gy. The DSBs calculated were in the range of values reported by other codes, but the error bars were very large. There was also a large dispersion in the calculations from other codes and in the experimental data.

The yield of SSB tended to decrease with the LET, while the yield of DSB tended to increase with the LET, giving rise to a strong negative dependence of the SSB to DSB ratio on LET. This can be attributed to the increasing proximity of the breaks with increasing LET values. Spatial effects were also evident in the protection of DNA by histones, with DNA loci in contact with the histones on the inside of the nucleosomes having the most protection, as expected intuitively.

## 5. Conclusions

Radiation-induced DNA damage is of fundamental importance in radiobiology, and its importance has been studied for several decades. However, the estimation of damage yields remains a challenging problem with many unknowns. Recently, there has seemed to be a renewed interest in the field, especially with improvements in our understanding of the epigenome, new DNA sequence-resolved experimental data from irradiated cells, the availability of increasingly powerful computing resources, and several updated simulation codes.

The code RITRACKS provides a framework for studying radiation-induced DNA damage. Although there are many other codes available, RITRACKS has a unique approach. The transport algorithm and the chemistry approach used in RITRACKS, to our knowledge, is not used by other codes. The cross sections model for DNA and amino acids, as well as the reaction rate constants, are also important simulation parameters unique to RITRACKS. For a problem with this level of complexity, having a diversity of codes and approaches is helpful to improve the understanding of radiation-induced DNA damages. The implementation of the simulation methods in coding require time. RITRACKS comprises over 1500 source files. To avoid bugs and crashes, coding is performed using standards and extensive testing.

This study provides the initial simulation results of DNA damage by RITRACKS 4.0. In general, the simulation results compared well with results from other codes. The results show that the presence of histones reduced the number of breaks by about 50% and that the breaks in DNA followed a periodic pattern of 10 bp. The display of the DNA breakage probability also shows that breakage tended to happen in the periphery of the nucleosome when histones were present. Taken together, these results show evidence of the histone protection of nucleosome-wrapped DNA.

In the future, we will expand the code to study radiation effects on more complex DNA structures and will compare the results from these simulations with RICC-seq and DNA damage mapping experiments.

## 6. New Technology

New technology report MSC-27643-1, RITRACKS, was filed to the NASA JSC Technology Transfer office.

## Figures and Tables

**Figure A1:**
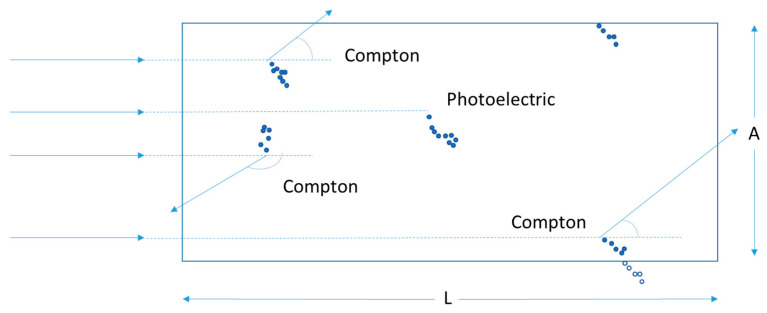
Configuration for photon irradiation. A volume with surface A and length L is irradiated with $N_{photons}$ photons of energy E. The photon can perform a photoelectric interaction—in which the photon is absorbed—and one (or more) electron(s) is (are) emitted. The photon can also perform a Compton interaction. In that case, the photon changes direction (including possibly going backwards), and its energy is reduced but usually still high enough to leave the irradiated volume. A Compton electron is emitted. The electrons make their own tracks in the volume (dots). If an electron has sufficient energy, it may leave the volume. When periodic boundary conditions are present, the electrons leaving the volume are put back on the other side with the same direction vector.

## Data Availability

The simulation results may be obtained by request to the corresponding author.

## Supplementary Materials

The following supporting information can be downloaded at https://www.mdpi.com/article/10.3390/biotech13020017/s1, Figure S1: Electron ionization cross sections for thymine orbitals; Figure S2: Total ionization cross sections for DNA constituents; Figure S3: Delta-G for selected reactions with DNA; Tables S1–S6: BEB parameters for DNA constituents; Tables S7–S26: BEB parameters for amino acids; Table S27: DEA cross sections for amino acids; Table S28: Reaction rate constant and reaction radii for radicals-DNA structure reactions; Table S29: Reaction rate constant and reaction radii for radicals—amino acids reactions; Table S30: Z scores for the DFT peak.

## Appendix A

Calculation of the number of photons for a given dose. In the energy range between 100 eV and 1 MeV, photons interact with the medium mainly through the Compton effect, resulting in the emission of a Compton electron and a photon with smaller energy. Photons of low energy are usually absorbed by photoelectric effect, resulting in the emission of a photoelectron. Therefore, photons lead to production of electrons in the medium. Pair production (creation of an electron and a positron) is also possible, but only for photons with energies greater than 1.022 MeV. Since the photons simulated have energies below 1 MeV, pair production will not be considered.

Consider a collimated beam of photons of energy E and initial fluence $\phi_{0}$ with total attenuation coefficient μ, as shown in Figure A1. The fluence of photons ϕ (i.e., number of photons per surface unit) at depth x is given by:

(A1) $$\phi (x)=\phi_{0}e^{-\mu x},$$

The fraction of photons interacting in the volume in the range [0,x] is given by:

(A2) $$f=\frac{\phi \left(0\right)-\phi \left(x\right)}{\phi \left(0\right)}=(1-e^{-\mu x}).$$

The average energy deposited by a photon of energy E during an interaction is given by $\bar{E}=(\mu_{ab}/\mu )E$, where $\mu_{ab}$ is the absorption coefficient. The irradiated volumes simulated in this work are much smaller than the mean free path of photons, except at low energies. Therefore, in a collimated photon beam, most photons will not interact at all in the volume, and those that do interact will only perform one interaction, although it is theoretically possible for a photon to perform more than one interaction in the target volume.

The dose (D) is the energy deposited in the volume divided by its mass. For a volume of surface A and length L irradiated by $N_{photons}$ photons, the dose can be calculated as:

(A3) $$D=\frac{E_{Dep}}{M}=\frac{N_{photons}*f*\bar{E}}{\rho LA}=\frac{(\phi_{0}A)(1-e^{-\mu L})(\mu_{ab}/\mu )E}{\rho LA}=\frac{\phi_{0}(1-e^{-\mu L})(\mu_{ab}/\mu )E}{\rho L},$$

where M is mass of the volume, ρ is the density, and $E_{Dep}$ is the energy deposited in the volume. The number of photons $N_{photons}$ is given by $\phi_{0}A$.

For a simulation, the requested dose is usually pre-determined, and the number of incident photons needed to get that dose is required. The fluence of photons corresponding to dose D is given by inversion of Equation (A3), i.e.,

(A4) $$\phi_{0}=\frac{D\rho L}{(1-e^{-\mu L})(\mu_{ab}/\mu )E}.$$

As for ions, the number of photons for each simulation history was determined by sampling of the Poisson distribution with $\lambda =\phi_{0}A$ as the average number of tracks.

The mean free path of photons was much larger than the typical volumes of micrometer sizes considered for the simulations, except at low photon energies. Therefore, only a very small fraction of the photons was expected to interact within the volume (Table A1).

**Table A1:** Irradiation of a volume, A = 10 µm × 10 µm, L = 5 µm, with photons, for a dose of 0.1 Gy.

Energy (keV)	μ (cm^2^/g)	$\mu_{ab}$ (cm^2^/g)	$\phi_{0}A$	$\phi \left(L\right)A$	$\phi_{0}A-\phi \left(L\right)A$	f (%)
1	4020	4020	360.85	48.35	312.5	86.6
2	566	566	633.93	477.68	156.25	24.6
3	179	179	1216.73	1112.56	104.17	8.56
5	39.6	39.5	3195.99	3133.33	62.66	1.96
10	4.87	4.72	13,257.65	13,225.40	32.25	0.24
20	0.676	0.505	61,891.64	61,870.72	20.92	0.0338
30	0.314	0.140	148,821.20	148,797.84	23.36	0.0157
50	0.204	0.0383	326,387.40	326,354.11	33.29	0.0102
100	0.165	0.0248	252,026.52	252,005.73	20.79	8.25 × 10^−3^
200	0.135	0.0295	105,935.77	105,928.62	7.15	6.74 × 10^−3^
300	0.118	0.0319	65,310.181	65,306.328	3.853	5.90 × 10^−3^
500	0.0966	0.0331	37,765.262	37,763.438	1.824	4.82 × 10^−3^
1000	0.0707	0.0310	20,161.646	20,160.933	0.713	3.53 × 10^−3^
