# Supplementary material for: Simulation of Radiation-Induced DNA Damage and Protection by Histones Using the Code RITRACKS
Source: BioTech (Basel). 2024 Jun 5;13(2):17. doi: 10.3390/biotech13020017 (PMC11201919; doi:10.3390/biotech13020017)
Supplement: Supplementary file 1 [file biotech-13-00017-s001.zip › biotech-2939072-supplementary.pdf]

Supplementary Material: Simulation of Radiation-Induced DNA Damage and Protection by Histones Using the Code RITRACKS

Ianik Plante, Devany W. West, Jason Weeks and Viviana I. Risca

1. Molecular orbitals

In this section, the parameters used in the BEB model are provided.

1.1 For DNA structure

Table S1. BEB parameters for Adenine ($C_5N_5H_4$) (69 electrons)

	No	U	B	N
Internal electrons (20)	1	601.40	426.64	2.00
	2	601.70	424.73	2.00
	3	601.30	424.56	2.00
	4	601.60	424.52	2.00
	5	601.60	424.23	2.00
	6	435.80	310.39	2.00
	7	435.70	309.74	2.00
	8	435.70	309.26	2.00
	9	435.80	308.68	2.00
	10	435.60	307.67	2.00
Valence electrons (49)	11	55.70	36.64	1.89
	12	57.09	34.96	1.81
	13	55.10	33.44	1.91
	14	54.33	31.74	1.88
	15	54.18	30.80	1.87
	16	48.27	27.68	2.04
	17	45.08	24.66	2.12
	18	46.16	23.80	2.02
	19	42.53	23.25	2.10
	20	39.62	20.95	2.12
	21	35.89	19.80	2.07
	22	31.70	18.87	2.13
	23	35.85	17.91	2.11
	24	35.05	17.18	2.15
	25	38.02	16.88	2.05
	26	39.92	16.63	1.82
	27	38.47	15.70	1.87
	28	43.08	14.85	1.92
	29	42.92	13.33	1.89
	30	42.80	13.12	1.84
	31	45.07	12.41	1.86
	32	44.99	11.58	1.84
	33	44.66	11.07	1.83
	34	45.75	10.65	1.85
	35	39.34	8.12	2.00

Table S2. BEB parameters for Cytosine ($C_4N_3OH_4$) (57 electrons)

	No	U	B	N
Internal electrons (16)	1	794.00	558.57	2.00
	2	601.50	425.51	2.00
	3	601.40	424.26	2.00
	4	601.60	424.06	2.00
	5	435.90	310.68	2.00
	6	435.90	309.69	2.00
	7	435.80	308.15	2.00
	8	435.60	306.24	2.00
Valence electrons (41)	9	67.91	39.32	1.97
	10	59.32	35.96	1.90
	11	56.03	34.87	1.91
	12	41.29	31.68	1.89
	13	45.62	28.83	1.96
	14	49.37	24.35	2.01
	15	45.80	24.27	2.09
	16	47.22	21.25	2.15
	17	38.94	20.60	2.00
	18	37.11	19.02	2.01
	19	25.79	18.03	2.13
	20	43.73	17.70	2.02
	21	48.40	17.07	2.00
	22	34.34	16.42	1.90
	23	44.08	16.16	1.96
	24	39.93	14.59	1.94
	25	48.70	13.76	1.91
	26	56.86	12.69	1.83
	27	49.86	11.94	1.85
	28	40.18	10.76	1.72
	29	42.94	9.41	1.83

Table S3. BEB parameters for Guanine ($C_2N_5OH_4$) (77 electrons)

	No	U	B	N
Internal electrons (22)	1	794.00	558.27	2.00
	2	601.60	426.44	2.00
	3	601.60	425.95	2.00
	4	601.60	424.66	2.00
	5	601.60	424.55	2.00
	6	601.60	424.38	2.00
	7	435.90	310.69	2.00
	8	435.90	309.94	2.00
	9	435.80	308.70	2.00
	10	435.80	308.00	2.00
	11	435.60	306.50	2.00
Valence electrons (55)	12	57.80	38.67	1.90
	13	55.30	37.70	1.94
	14	54.12	37.03	1.90
	15	52.96	33.77	1.97
	16	57.37	33.64	1.89
	17	56.46	32.48	1.85
	18	50.67	28.77	2.02
	19	45.95	25.14	2.02
	20	49.02	24.69	1.87
	21	45.39	23.18	2.15
	22	40.13	22.44	2.13
	23	43.27	20.75	1.82
	24	47.56	20.52	2.01
	25	43.93	17.45	2.14
	26	46.47	16.56	2.19
	27	31.80	15.78	2.26
	28	49.35	15.26	2.10
	29	44.63	14.48	1.98
	30	63.78	14.34	1.96
	31	35.03	14.33	1.97
	32	41.05	13.23	1.97
	33	51.10	10.88	1.90
	34	41.58	10.63	1.83
	35	48.79	9.99	1.76
	36	63.50	9.80	1.82
	37	54.22	9.38	1.84
	38	43.57	9.71	1.79
	39	41.75	7.77	2.03

Table S4. BEB parameters for Thymine ($C_5N_2O_2H_2$) (62 electrons)

	No	U	B	N
Internal electrons (18)	1	794.1	559.41	2.00
	2	794.1	559.18	2.00
	3	601.5	425.60	2.00
	4	601.5	425.48	2.00
	5	435.9	311.36	2.00
	6	435.9	310.43	2.00
	7	435.8	308.00	2.00
	8	435.7	306.41	2.00
	9	435.8	305.80	2.00
Valence electrons (44)	10	66.26	40.06	2.00
	11	71.74	39.14	2.04
	12	63.05	36.16	1.86
	13	57.89	34.56	1.85
	14	44.95	30.00	2.30
	15	43.96	26.22	2.35
	16	47.64	25.14	2.11
	17	40.64	24.85	2.15
	18	41.92	21.32	2.18
	19	39.28	20.94	2.02
	20	36.32	19.40	1.86
	21	44.53	18.70	2.05
	22	55.89	18.56	1.92
	23	54.72	17.59	1.99
	24	39.88	16.62	2.01
	25	46.93	16.15	2.03
	26	39.89	15.44	1.78
	27	47.30	13.96	1.83
	28	59.96	13.15	1.86
	29	54.12	12.31	2.07
	30	60.23	12.10	1.78
	31	40.38	9.27	1.97

Table S5. BEB parameters for Sugar (C_5H_7O) (45 electrons)

	No	U	B	N
Internal electrons (12)	1	794.00	559.43	2.00
	2	435.90	307.36	2.00
	3	435.90	306.98	2.00
	4	435.90	306.22	2.00
	5	435.90	305.47	2.00
	6	435.90	305.43	2.00
Valence electrons (33)	7	75.43	38.42	1.94
	8	21.73	34.03	2.20
	9	44.28	29.58	1.92
	10	42.52	27.64	2.06
	11	39.26	22.93	2.00
	12	39.86	21.96	1.97
	13	41.84	20.14	1.80
	14	37.46	19.00	1.93
	15	37.00	17.94	1.71
	16	30.83	16.53	2.06
	17	36.42	15.67	1.99
	18	35.56	13.94	1.96
	19	43.83	13.14	1.91
	20	30.15	12.97	1.90
	21	24.09	12.36	1.96
	22	46.29	11.49	1.96
	23	31.98	9.13	1.77

Table S6. BEB parameters for Phosphate (PO_4^{2-}) (49 electrons)

	No	U	B	N
Internal electrons (18)	1	2939.00	2179.19	2.00
	2	794.00	559.71	2.00
	3	794.00	556.81	2.00
	4	794.00	556.37	2.00
	5	794.00	555.90	2.00
	6	445.00	207.24	2.00
	7	139.10	150.01	2.00
	8	139.10	149.99	2.00
	9	139.10	149.95	2.00
Valence electrons (31)	10	81.67	38.23	1.71
	11	78.61	34.02	1.96
	12	81.61	32.41	1.93
	13	81.74	31.03	2.00
	14	65.89	21.22	1.82
	15	62.70	17.42	1.86
	16	60.29	15.76	2.00
	17	59.56	15.56	2.07
	18	60.15	13.47	2.08
	19	63.22	12.74	1.97
	20	63.47	11.61	1.93
	21	66.90	11.09	1.91
	22	66.60	10.48	1.90
	23	66.01	10.33	1.97
	24	66.91	9.84	1.98
	25	67.10	9.62	1.92

1.2 For amino acids

Table S7. BEB parameters for Alanine (C_3ONH_5) (38 electrons)

	No	U	B	N
Internal electrons (10)	1	794.10	559.95	2.00
	2	601.60	423.10	2.00
	3	435.60	310.77	2.00
	4	435.60	307.30	2.00
	5	435.60	305.59	2.00
Valence electrons (28)	6	52.90	32.85	2.01
	7	43.85	27.87	2.07
	8	40.84	24.83	2.04
	9	48.43	19.75	2.23
	10	50.67	19.56	1.81
	11	46.16	18.09	1.84
	12	30.56	16.17	1.96
	13	40.01	15.77	1.80
	14	33.62	15.54	1.77
	15	32.81	14.83	2.18
	16	23.96	13.84	2.12
	17	58.31	13.48	1.86
	18	53.02	12.44	2.32
	19	43.95	10.84	1.99

Table S8. BEB parameters for Arginine ($C_6N_4OH_{12}$) (84 electrons)

	No	U	B	N
Internal electrons (22)	1	794.10	559.54	2.00
	2	601.60	423.87	2.00
	3	601.60	423.76	2.00
	4	601.60	422.86	2.00
	5	601.60	422.73	2.00
	6	435.60	310.28	2.00
	7	435.60	309.49	2.00
	8	435.60	307.03	2.00
	9	435.60	306.60	2.00
	10	435.60	305.49	2.00
	11	435.60	305.13	2.00
Valence electrons (62)	12	54.56	35.06	1.96
	13	52.55	32.84	1.95
	14	55.22	31.91	1.78
	15	51.42	30.89	1.94
	16	44.46	28.61	1.89
	17	42.52	26.85	2.11
	18	40.20	24.65	2.06
	19	39.08	22.47	1.92
	20	39.68	21.62	1.94
	21	38.55	20.81	1.90
	22	38.99	19.87	2.17
	23	42.28	19.26	2.18
	24	30.95	18.61	2.27
	25	47.79	17.91	2.07
	26	36.23	17.46	1.99
	27	36.05	17.24	1.77
	28	33.48	16.36	2.09
	29	32.99	16.13	1.88
	30	38.18	15.52	1.81
	31	34.36	15.15	2.03
	32	32.91	14.84	2.01
	33	35.73	14.12	2.11
	34	33.02	13.58	2.01
	35	52.70	12.99	1.96
	36	38.92	12.98	2.17
	37	33.25	12.79	2.19
	38	51.98	11.95	2.34
	39	39.42	11.27	2.03
	40	47.37	10.78	1.76
	41	45.02	10.43	1.92
	42	43.06	9.59	1.79

Table S9. BEB parameters for Asparagine ($C_4N_2O_2H_6$) (60 electrons)

	No	U	B	N
Internal electrons (16)	1	794.10	559.72	2.00
	2	794.10	558.21	2.00
	3	601.60	423.65	2.00
	4	601.60	423.23	2.00
	5	435.60	310.57	2.00
	6	435.60	309.19	2.00
	7	435.60	307.45	2.00
	8	435.60	306.28	2.00
Valence electrons (44)	9	72.84	38.70	2.24
	10	53.30	33.05	1.91
	11	54.73	32.69	1.98
	12	44.69	28.58	1.97
	13	43.74	25.63	2.07
	14	47.84	22.37	1.73
	15	44.18	21.20	1.72
	16	46.81	20.28	1.86
	17	38.39	19.86	2.08
	18	47.32	19.18	2.26
	19	43.38	18.41	2.00
	20	43.75	17.89	2.16
	21	38.03	16.58	2.15
	22	45.51	16.10	2.21
	23	38.23	15.93	1.81
	24	43.21	15.32	1.99
	25	33.26	14.96	1.99
	26	39.72	13.57	2.18
	27	56.35	12.41	2.31
	28	54.33	11.47	1.82
	29	49.26	10.88	1.91
	30	59.62	9.80	1.65

Table S10. BEB parameters for Aspartic acid ($C_4NO_3H_5$) (60 electrons)

	No	U	B	N
Internal electrons (16)	1	794.1	560.94	2.00
	2	794.1	559.79	2.00
	3	794.1	559.27	2.00
	4	601.6	423.26	2.00
	5	435.6	310.64	2.00
	6	435.6	310.36	2.00
	7	435.6	307.53	2.00
	8	435.6	306.53	2.00
Valence electrons (44)	9	71.17	40.44	2.05
	10	75.84	38.18	1.87
	11	74.91	37.34	1.71
	12	52.62	33.09	1.92
	13	45.1	28.94	2.10
	14	43.89	26.01	2.20
	15	48.54	22.91	2.09
	16	41.53	19.96	2.04
	17	52.36	19.26	2.24
	18	51.11	18.75	2.20
	19	47.69	17.83	2.18
	20	48.61	17.55	1.90
	21	39.44	16.24	2.23
	22	43.11	16.09	1.61
	23	37.62	15.52	2.13
	24	45.21	15.46	2.12
	25	36.87	14.38	2.17
	26	54.68	13.54	1.86
	27	60.72	12.67	1.74
	28	53.48	12.48	2.06
	29	50.93	11.19	1.79
	30	55.74	10.84	1.79

Table S11. BEB parameters for Cysteine (SC_3NOH_5) (54 electrons)

	No	U	B	N
Internal electrons (20)	1	3356.00	2502.41	2.00
	2	794.10	560.12	2.00
	3	601.60	423.15	2.00
	4	435.60	310.96	2.00
	5	435.60	307.62	2.00
	6	435.60	307.00	2.00
	7	524.39	244.06	2.00
	8	505.78	240.00	2.00
	9	499.89	180.95	2.00
	10	499.89	180.88	2.00
Valence electrons (34)	11	52.95	33.22	1.87
	12	51.88	29.10	1.88
	13	52.78	26.97	2.08
	14	48.97	23.68	1.92
	15	37.28	22.11	1.63
	16	46.71	19.99	2.39
	17	47.54	18.27	2.12
	18	44.04	17.48	1.78
	19	34.12	16.90	1.89
	20	39.62	16.38	1.79
	21	44.71	15.84	1.59
	22	33.15	15.00	2.03
	23	34.62	14.36	2.26
	24	52.55	13.31	2.60
	25	50.91	12.05	2.32
	26	45.29	11.08	1.82
	27	50.04	10.09	2.03

Table S12. BEB parameters for Glutamic acid ($C_5NO_3H_7$) (68 electrons)

	No	U	B	N
Internal electrons (18)	1	794.10	561.15	2.00
	2	794.10	559.99	2.00
	3	794.10	559.68	2.00
	4	601.60	423.12	2.00
	5	435.60	310.84	2.00
	6	435.60	310.57	2.00
	7	435.60	307.34	2.00
	8	435.60	305.91	2.00
	9	435.60	305.83	2.00
Valence electrons (50)	10	71.89	40.75	2.07
	11	76.16	37.86	1.99
	12	52.50	32.95	1.93
	13	43.91	29.45	1.95
	14	44.77	27.02	2.10
	15	43.81	24.59	2.05
	16	45.61	22.30	1.70
	17	42.98	21.71	1.91
	18	50.17	19.95	2.12
	19	57.28	19.40	2.06
	20	53.78	18.74	2.21
	21	43.43	18.22	1.93
	22	49.18	17.96	1.91
	23	44.62	16.44	2.12
	24	43.91	16.27	1.74
	25	37.59	16.06	1.98
	26	31.66	15.18	2.09
	27	44.50	15.1	1.94
	28	44.81	140	1.93
	29	35.15	13.51	2.27
	30	58.60	13.48	1.97
	31	50.24	13.01	1.92
	32	53.51	12.49	2.31
	33	46.69	12.24	1.90
	34	44.72	10.81	1.89

Table S13. BEB parameters for Glutamine ($C_5N_2O_2H_8$) (68 electrons)

	No	U	B	N
Internal electrons (18)	1	794.10	560.01	2.00
	2	794.10	559.50	2.00
	3	601.60	423.41	2.00
	4	601.60	423.25	2.00
	5	435.60	310.86	2.00
	6	435.60	309.80	2.00
	7	435.60	307.47	2.00
	8	435.60	306.14	2.00
	9	435.60	306.01	2.00
Valence electrons (50)	10	71.72	39.08	1.99
	11	53.53	33.24	1.88
	12	52.94	32.83	1.98
	13	43.64	29.16	1.97
	14	43.99	27.18	2.11
	15	42.96	24.07	1.99
	16	40.91	21.30	2.17
	17	42.93	20.11	2.20
	18	63.71	19.67	1.79
	19	35.44	19.29	2.23
	20	43.62	18.72	2.03
	21	40.45	18.17	2.15
	22	49.15	17.91	1.85
	23	33.61	16.89	1.87
	24	35.36	16.08	1.83
	25	37.13	15.85	1.97
	26	43.39	15.38	2.01
	27	31.43	14.41	2.16
	28	41.70	14.00	1.94
	29	35.11	13.62	2.26
	30	53.07	13.47	1.77
	31	53.37	12.48	2.23
	32	52.15	12.02	1.93
	33	49.27	11.33	1.79
	34	45.21	10.88	1.91

Table S14. BEB parameters for Glycine (C_2NOH_3) (30 electrons)

	No	U	B	N
Internal electrons (8)	1	794.10	559.92	2.00
	2	601.60	423.11	2.00
	3	435.60	310.78	2.00
	4	435.60	307.12	2.00
Valence electrons (22)	5	53.50	32.52	1.87
	6	44.84	26.79	2.12
	7	54.49	19.64	2.37
	8	46.46	19.34	1.92
	9	39.16	18.49	2.02
	10	43.11	17.07	1.68
	11	38.97	16.52	1.88
	12	41.38	15.64	1.67
	13	32.27	14.91	1.91
	14	59.91	12.68	2.65
	15	42.54	10.96	1.91

Table S15. BEB parameters for Histidine ($C_6N_3OH_7$) (72 electrons)

	No	U	B	N
Internal electrons (20)	1	794.1	559.97	2.00
	2	601.6	425.21	2.00
	3	601.6	424.06	2.00
	4	601.6	422.93	2.00
	5	435.6	310.88	2.00
	6	435.6	308.49	2.00
	7	435.6	307.48	2.00
	8	435.6	307.42	2.00
	9	435.6	306.74	2.00
	10	435.6	306.27	2.00
Valence electrons (52)	11	54.52	36.18	1.94
	12	51.81	33.43	1.95
	13	54.76	31.76	1.93
	14	45.37	29.12	2.00
	15	43.95	27.99	2.06
	16	42.2	24.97	2.04
	17	39.81	22.82	2.13
	18	38.89	21.43	2.10
	19	39.72	20.24	2.15
	20	66.91	19.68	1.85
	21	38.54	19.28	2.10
	22	40.29	18.46	2.05
	23	42.98	17.78	1.62
	24	32.97	17.02	1.99
	25	34.95	16.85	2.15
	26	36.8	15.81	1.98
	27	33.95	15.58	1.93
	28	35.57	14.91	2.10
	29	39.14	14.13	2.03
	30	27.8	14.07	2.12
	31	61.76	13.57	1.73
	32	52.99	12.6	2.24
	33	44.13	12.37	1.98
	34	44.99	11.32	1.89
	35	44.17	10.93	1.86
	36	34.63	9.07	2.07

Table S16. BEB parameters for Isoleucine (C_6NOH_{11}) (62 electrons)

	No	U	B	N
Internal electrons (16)	1	794.10	561.65	2.00
	2	601.60	423.53	2.00
	3	435.60	312.47	2.00
	4	435.60	307.85	2.00
	5	435.60	305.99	2.00
	6	435.60	305.58	2.00
	7	435.60	305.30	2.00
	8	435.60	302.94	2.00
Valence electrons (46)	9	69.62	40.92	2.14
	10	51.84	33.64	1.88
	11	44.35	29.97	1.84
	12	41.31	27.43	1.59
	13	40.39	25.91	1.95
	14	41.31	22.11	1.58
	15	45.03	20.78	2.11
	16	33.17	20.32	1.75
	17	49.48	19.33	2.04
	18	33.69	17.59	2.12
	19	42.53	17.26	1.68
	20	5.450	16.76	2.14
	21	37.57	15.81	2.09
	22	40.04	15.57	2.03
	23	33.17	15.11	2.14
	24	28.59	14.72	2.20
	25	33.19	14.52	2.05
	26	32.00	13.59	2.18
	27	26.86	13.31	2.18
	28	21.91	12.43	2.16
	29	29.95	11.84	1.96
	30	42.32	11.08	1.87
	31	23.76	5.13	2.32

Table S17. BEB parameters for Leucine (C_6NOH_{11}) (62 electrons)

	No	U	B	N
Internal electrons (16)	1	794.10	561.66	2.00
	2	601.60	423.51	2.00
	3	435.60	312.49	2.00
	4	435.60	307.87	2.00
	5	435.60	305.84	2.00
	6	435.60	305.49	2.00
	7	435.60	304.96	2.00
	8	435.60	302.94	2.00
Valence electrons (46)	9	48.11	40.92	2.16
	10	48.23	33.52	1.87
	11	42.60	29.96	1.86
	12	35.90	27.32	1.83
	13	33.98	24.10	1.91
	14	33.92	22.63	1.72
	15	40.91	20.58	1.99
	16	30.91	20.08	1.85
	17	42.82	19.43	2.01
	18	36.81	17.52	2.10
	19	35.91	16.90	2.03
	20	37.12	16.59	1.75
	21	38.69	16.42	1.88
	22	40.02	15.49	2.11
	23	34.62	14.79	2.02
	24	37.41	14.51	2.09
	25	31.65	13.99	2.08
	26	31.73	13.56	1.96
	27	32.55	13.38	2.04
	28	27.13	12.32	2.11
	29	30.10	11.98	1.87
	30	41.19	10.91	1.89
	31	25.21	5.08	2.88

Table S18. BEB parameters for Lysine ($C_6N_2OH_{12}$) (70 electrons)

	No	U	B	N
Internal electrons (18)	1	794.10	559.99	2.00
	2	601.60	423.18	2.00
	3	601.60	422.74	2.00
	4	435.60	310.80	2.00
	5	435.60	307.25	2.00
	6	435.60	306.21	2.00
	7	435.60	305.61	2.00
	8	435.60	305.49	2.00
	9	435.60	305.40	2.00
Valence electrons (52)	10	52.80	33.09	1.82
	11	50.58	32.23	1.87
	12	42.87	29.26	1.87
	13	44.62	27.43	1.96
	14	40.87	25.62	2.03
	15	38.94	23.43	1.96
	16	42.95	22.17	1.57
	17	35.63	20.70	2.08
	18	45.28	19.99	2.23
	19	48.74	18.23	2.20
	20	33.69	17.98	2.12
	21	37.12	17.63	1.90
	22	44.79	17.29	1.70
	23	30.25	16.41	2.06
	24	33.34	16.24	1.89
	25	36.94	16.01	1.56
	26	37.59	15.43	2.14
	27	29.71	14.65	1.96
	28	32.36	14.53	2.23
	29	29.21	14.21	2.10
	30	43.97	12.99	2.42
	31	28.19	12.69	2.15
	32	33.66	12.40	2.23
	33	39.71	12.04	2.31
	34	44.01	10.90	1.80
	35	39.72	10.41	1.85

Table S19. BEB parameters for Methionine (SC_5NOH_9) (70 electrons)

	No	U	B	N
Internal electrons (24)	1	3356.00	2502.16	2.00
	2	794.10	559.95	2.00
	3	601.60	423.19	2.00
	4	435.60	310.79	2.00
	5	435.60	307.24	2.00
	6	435.60	306.76	2.00
	7	435.60	306.52	2.00
	8	435.60	305.94	2.00
	9	524.39	243.80	2.00
	10	507.78	180.73	2.00
	11	499.89	180.68	2.00
	12	499.89	180.62	2.00
Valence electrons (46)	13	52.69	33.13	1.85
	14	51.27	30.06	1.92
	15	47.33	27.85	2.01
	16	41.74	26.47	2.02
	17	43.26	24.09	2.01
	18	45.99	22.35	1.60
	19	41.07	21.18	2.03
	20	45.22	19.91	2.21
	21	44.16	18.39	2.15
	22	46.32	17.37	1.76
	23	30.84	16.70	2.04
	24	41.32	16.01	1.47
	25	27.19	15.92	2.04
	26	27.96	15.43	2.12
	27	40.19	15.02	2.13
	28	28.75	14.42	1.95
	29	28.24	14.17	2.15
	30	60.99	13.51	2.00
	31	29.61	12.95	2.11
	32	56.39	12.50	2.46
	33	42.55	11.61	2.03
	34	43.30	10.91	1.85
	35	47.05	9.46	2.08

Table S20. BEB parameters for Phenylalanine (C_9NOH_9) (78 electrons)

	No	U	B	N
Internal electrons (22)	1	794.1	560.13	2.00
	2	601.6	423.3	2.00
	3	435.6	311.03	2.00
	4	435.6	307.5	2.00
	5	435.6	306.48	2.00
	6	435.6	306.28	2.00
	7	435.6	306.26	2.00
	8	435.6	306.19	2.00
	9	435.6	306.18	2.00
	10	435.6	306.16	2.00
	11	435.6	306.15	2.00
Valence electrons (56)	12	52.27	33.25	1.85
	13	44.39	31.32	1.89
	14	43.52	28.94	1.90
	15	40.81	27.6	1.96
	16	44.38	27.35	2.02
	17	42.17	25.01	1.99
	18	38.41	22.68	1.95
	19	43.9	22.23	1.58
	20	37.69	21.58	2.01
	21	48.31	20.01	2.36
	22	37.21	18.87	2.11
	23	43.92	18.12	2.05
	24	44.89	17.64	1.85
	25	27.56	17.28	2.05
	26	32.24	17.23	1.89
	27	35.59	16.43	1.64
	28	34.62	16.13	1.80
	29	28.82	16.01	1.99
	30	31.77	15.68	2.07
	31	36.44	15.4	2.12
	32	26.32	14.27	2.12
	33	42.66	13.51	2.34
	34	32.8	13.3	2.04
	35	26.55	13.16	2.10
	36	52.95	12.66	2.66
	37	44.24	11.16	1.77
	38	33.21	9.23	1.93
	39	32.71	8.99	1.96

Table S21. BEB parameters for Proline (C_5NOH_7) (52 electrons)

	No	U	B	N
Internal electrons (14)	1	794.10	560.25	2.00
	2	601.60	423.07	2.00
	3	435.60	310.88	2.00
	4	435.60	307.14	2.00
	5	435.60	306.30	2.00
	6	435.60	306.14	2.00
	7	435.60	305.69	2.00
Valence electrons (38)	8	52.64	34.11	1.94
	9	43.54	28.75	1.95
	10	42.61	27.91	2.13
	11	39.64	24.04	2.15
	12	37.01	22.05	2.04
	13	38.96	21.72	1.85
	14	37.78	20.63	2.20
	15	45.20	19.28	2.14
	16	46.30	18.50	2.21
	17	34.45	17.37	1.83
	18	30.98	16.11	2.11
	19	35.42	14.59	2.01
	20	45.36	14.26	2.05
	21	44.74	14.00	1.36
	22	44.38	13.78	1.91
	23	34.73	13.19	1.97
	24	28.50	13.10	2.03
	25	43.69	12.50	2.07
	26	38.74	10.26	2.04

Table S22. BEB parameters for Serine ($C_3NO_2H_5$) (46 electrons)

	No	U	B	N
Internal electrons (12)	1	794.10	560.03	2.00
	2	794.10	559.56	2.00
	3	601.60	423.17	2.00
	4	435.60	310.88	2.00
	5	435.60	307.42	2.00
	6	435.60	307.40	2.00
Valence electrons (34)	7	71.98	37.88	1.94
	8	53.43	32.86	2.01
	9	45.39	27.94	2.11
	10	44.73	24.72	2.13
	11	49.02	20.06	2.17
	12	57.31	19.67	1.86
	13	42.21	18.97	2.10
	14	42.39	18.48	2.12
	15	48.41	16.98	1.61
	16	38.99	16.43	2.07
	17	40.07	15.86	1.71
	18	44.66	15.13	2.11
	19	35.22	13.97	2.03
	20	56.44	13.54	1.89
	21	54.21	13.00	1.99
	22	58.09	12.46	2.15
	23	44.26	10.95	2.00

Table S23. BEB parameters for Threonine ($C_4NO_2H_7$) (54 electrons)

	No	U	B	N
Internal electrons (14)	1	794.10	559.72	2.00
	2	794.10	559.15	2.00
	3	601.60	423.21	2.00
	4	435.60	310.64	2.00
	5	435.60	307.47	2.00
	6	435.60	307.29	2.00
	7	435.60	305.62	2.00
Valence electrons (40)	8	73.28	37.53	1.84
	9	53.84	33.02	1.94
	10	45.54	28.37	2.08
	11	41.18	26.49	2.09
	12	42.77	23.56	2.03
	13	50.95	19.85	2.08
	14	46.62	19.50	2.00
	15	43.93	18.45	2.23
	16	42.60	18.30	1.96
	17	40.40	17.35	2.05
	18	41.93	16.59	1.80
	19	41.88	15.82	1.72
	20	37.13	14.89	2.08
	21	29.87	14.73	2.01
	22	27.40	14.20	2.08
	23	49.03	13.53	1.91
	24	52.20	13.00	1.85
	25	60.89	12.59	2.07
	26	47.36	11.94	2.19
	27	44.41	11.00	1.96

Table S24. BEB parameters for Tryptophan ($C_{11}N_2O_2H_{13}$) (108 electrons)

	No	U	B	N
Internal electrons (30)	1	794.10	561.57	2.00
	2	794.10	560.25	2.00
	3	601.60	425.39	2.00
	4	601.60	423.19	2.00
	5	435.60	310.90	2.00
	6	435.60	307.93	2.00
	7	435.60	307.27	2.00
	8	435.60	307.14	2.00
	9	435.60	307.09	2.00
	10	435.60	306.94	2.00
	11	435.60	306.87	2.00
	12	435.60	306.44	2.00
	13	435.60	306.30	2.00
	14	435.60	306.19	2.00
	15	435.60	306.11	2.00
Valence electrons (78)	16	72.05	41.08	1.97
	17	76.43	38.16	1.91
	18	52.67	33.83	1.87
	19	50.10	33.02	1.89
	20	44.67	30.12	1.93
	21	43.19	28.83	1.94
	22	42.70	27.77	1.96
	23	43.63	26.69	2.03
	24	43.30	25.72	2.00
	25	42.40	24.39	2.06
	26	41.45	22.25	2.09
	27	42.86	22.06	2.13
	28	35.91	21.51	2.09
	29	37.61	20.68	2.07
	30	48.74	19.81	2.04
	31	49.16	19.57	2.00
	32	34.31	19.10	2.14
	33	45.30	18.22	2.00
	34	39.55	17.78	2.05
	35	42.41	17.45	2.06
	36	34.10	17.18	2.12
	37	29.31	16.59	2.14
	38	38.51	16.26	2.00
	39	30.16	16.00	2.05
	40	35.18	15.88	2.10
	41	35.41	15.65	2.05
	42	32.53	15.27	2.03
	43	35.42	14.77	1.96
	44	31.28	14.54	2.13

	45	37.73	14.19	1.94
	46	42.06	13.62	1.83
	47	49.27	12.91	2.03
	48	36.14	12.57	1.94
	49	40.42	11.92	1.95
	50	35.18	11.47	1.89
	51	38.96	10.92	1.78
	52	35.06	9.07	1.93
	53	23.60	7.36	1.93
	54	25.32	6.59	1.96

Table S25. BEB parameters for Tyrosine ($C_9NO_2H_9$) (84 electrons)

	No	U	B	N
Internal electrons (24)	1	794.10	560.18	2.00
	2	794.10	560.01	2.00
	3	601.60	423.08	2.00
	4	435.60	310.91	2.00
	5	435.60	308.05	2.00
	6	435.60	307.43	2.00
	7	435.60	306.51	2.00
	8	435.60	306.40	2.00
	9	435.60	306.36	2.00
	10	435.60	306.33	2.00
	11	435.60	306.31	2.00
	12	435.60	306.14	2.00
Valence electrons (62)	13	73.49	38.60	1.90
	14	52.14	33.42	1.84
	15	45.37	31.31	1.92
	16	44.32	28.89	1.90
	17	41.42	27.91	1.98
	18	44.65	27.39	2.04
	19	44.33	24.91	2.00
	20	39.85	23.19	2.01
	21	46.20	22.25	1.59
	22	39.59	21.92	1.97
	23	42.66	20.19	2.18
	24	45.64	19.92	2.30
	25	44.33	18.38	1.99
	26	51.68	18.34	2.11
	27	33.74	18.06	1.96
	28	39.20	17.49	1.87
	29	45.87	17.09	1.87
	30	30.38	16.48	1.94
	31	28.59	16.31	2.16
	32	37.62	16.11	1.84
	33	44.13	16.08	1.83
	34	35.91	15.27	1.84
	35	38.93	14.68	1.94
	36	37.83	14.32	2.22
	37	37.97	13.86	2.27
	38	35.12	13.20	2.10
	39	39.85	12.94	1.95
	40	55.43	12.56	2.77
	41	42.79	10.89	1.80
	42	33.54	9.40	1.91
	43	30.75	8.79	2.03

Table S26. BEB parameters for Valine (C_5NOH_9) (54 electrons)

	No	U	B	N
Internal electrons (14)	1	794.10	559.99	2.00
	2	601.60	423.09	2.00
	3	435.60	310.83	2.00
	4	435.60	307.23	2.00
	5	435.60	305.86	2.00
	6	435.60	305.52	2.00
	7	435.60	304.85	2.00
Valence electrons (40)	8	41.47	33.05	1.87
	9	40.41	29.39	1.90
	10	40.23	26.55	2.09
	11	35.49	25.23	1.99
	12	37.50	22.86	1.68
	13	34.00	21.36	1.84
	14	30.78	19.56	2.17
	15	33.69	17.53	1.76
	16	32.50	16.94	2.14
	17	28.59	16.59	1.78
	18	29.31	16.14	1.96
	19	26.87	15.40	1.99
	20	25.15	14.75	2.05
	21	23.00	14.04	2.16
	22	28.58	13.91	2.09
	23	28.05	13.35	2.16
	24	30.45	12.67	2.24
	25	28.64	12.52	2.16
	26	24.60	12.09	2.11
	27	31.81	10.70	1.95

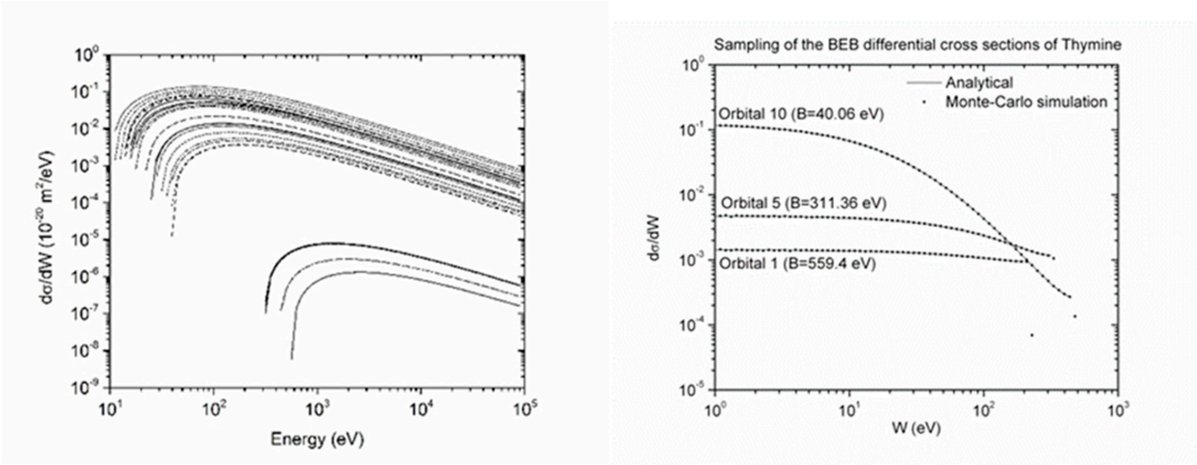

Figure S1. Left: Electron ionization cross sections for thymine orbitals (Equation 4 in the main text). Each line corresponds to a different molecular orbital. As the ionization is only possible for energies greater than the ionization potential, the cross section is 0 for energies below the corresponding ionization potential. The lower curves with high ionization potential are inner orbitals. Right: Sampling of the BEB differential cross section of thymine for T= 1 keV (squares), and comparison with the predicted analytical values (Equation 3 in the main text) (lines).

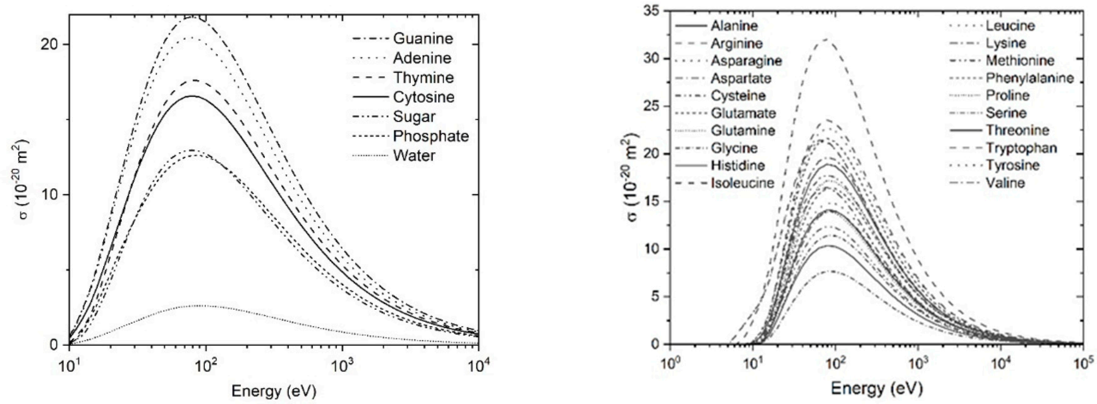

Figure S2. Left: Total Ionization Cross Sections (TICS) for adenine, cytosine, guanine, thymine, and for the sugar and phosphate groups, as a function of the electron energy. The total ionization cross section for water is shown for comparison. Right: TICS for the amino acids used in RITRACKS, as a function of the electron energy.

2. Dissociative Electron Attachment (DEA) cross sections

The DEA cross sections for amino acids are taken from Scheer et al., 2007. The DEA cross sections are provided for alanine, glycine, proline, phenylalanine, and tryptophan. In the following table, the DEA cross section taken for the missing amino acids are provided.

Table S27. DEA cross sections for amino acids

Amino acid	Cross section used
Alanine	Alanine
Arginine	Alanine
Asparagine	Alanine
Aspartic acid	Alanine
Cysteine	Alanine
Glutamic acid	Alanine
Glutamine	Alanine
Glycine	Glycine
Histidine	Proline
Isoleucine	Alanine
Leucine	Alanine
Lysine	Alanine
Methionine	Alanine
Phenylalanine	Phenylalanine
Proline	Proline
Serine	Glycine
Threonine	Glycine
Tryptophan	Tryptophan
Tyrosine	Phenylalanine
Valine	Alanine

3. Chemical reactions

3.1 With DNA structures

In this section, the rate constants and reaction radii of the chemical reactions between the radical species and DNA structure are given.

Table S28. Reaction rate constant and reaction radii for radicals-DNA structure reactions.

Structure	$H^{\bullet}$		$^{\bullet}OH$		e^{-}_{aq}	
	$k(M^{-1}s^{-1})$	$R\ (nm)$	$k(M^{-1}s^{-1})$	$R\ (nm)$	$k(M^{-1}s^{-1})$	$R\ (nm)$
Adenine	1.0×10^{8}	0.0019	6.1×10^{9}	0.288	1.34×10^{10}	0.396
Cytosine	9.2×10^{7}	0.0017	6.1×10^{9}	0.288	1.54×10^{10}	0.455
Guanine	-	-	9.2×10^{9}	0.425	1.59×10^{10}	0.47
Thymine	5.7×10^{8}	0.011	6.4×10^{9}	0.302	1.79×10^{10}	0.5287
Sugar	2.9×10^{7}	0.0006	1.8×10^{9}	0.085	-	-
Phosphate	2.9×10^{7}	0.0006	1.8×10^{9}	0.085	-	-

3.2 With amino acids

Table S29. Reaction rate constant and reaction radii for radicals – amino acids reactions.

Structure	$H^{\bullet}$		$^{\bullet}OH$		e^{-}_{aq}	
	$k(M^{-1}s^{-1})$	$R\ (nm)$	$k(M^{-1}s^{-1})$	$R\ (nm)$	$k(M^{-1}s^{-1})$	$R\ (nm)$
Alanine	2.7×10^{5}	5.09×10^{-6}	4.3×10^{8}	2.58×10^{-2}	9.00×10^{6}	2.43×10^{-4}
Arginine	4.5×10^{6}	8.50×10^{-5}	3.5×10^{9}	0.21	5.00×10^{7}	1.35×10^{-3}
Asparagine	4.3×10^{5}	8.11×10^{-6}	4.9×10^{7}	2.94×10^{-3}	1.50×10^{8}	4.05×10^{-3}
Aspartic acid	7.0×10^{5}	1.32×10^{-5}			6.00×10^{8}	1.62×10^{-2}
Cysteine	1.8×10^{9}	3.39×10^{-2}	1.90×10^{10}	1.14	8.50×10^{9}	0.23
Glutamic acid	3.0×10^{6}	5.66×10^{-5}	1.60×10^{8}	9.61×10^{-3}	2.00×10^{7}	5.39×10^{-4}
Glutamine			5.40×10^{8}	3.24×10^{-2}		
Glycine	7.1×10^{5}	1.34×10^{-6}	1.70×10^{7}	1.02×10^{-3}	1.00×10^{7}	2.70×10^{-4}
Histidine	2.3×10^{8}	4.34×10^{-3}	4.80×10^{9}	0.29	9.00×10^{8}	2.43×10^{-2}
Isoleucine	1.6×10^{7}	3.02×10^{-4}	1.80×10^{9}	0.11	1.00×10^{7}	2.70×10^{-4}
Leucine	1.6×10^{7}	3.02×10^{-4}	2.00×10^{9}	0.12	1.00×10^{7}	2.70×10^{-4}
Lysine	1.5×10^{6}	2.83×10^{-5}	3.50×10^{8}	2.10×10^{-2}	2.00×10^{7}	5.39×10^{-4}
Methionine	3.5×10^{8}	6.61×10^{-3}	7.40×10^{9}	0.44	4.50×10^{7}	1.21×10^{-3}
Phenylalanine	7.1×10^{8}	1.34×10^{-2}	6.50×10^{9}	0.39	1.20×10^{8}	3.24×10^{-3}
Proline	7.0×10^{5}	1.32×10^{-5}	3.10×10^{8}	1.86×10^{-2}	2.00×10^{7}	5.39×10^{-4}
Serine	1.2×10^{6}	2.27×10^{-5}	3.20×10^{8}	1.92×10^{-2}	3.00×10^{7}	8.09×10^{-4}
Threonine	7.0×10^{6}	1.32×10^{-4}	5.10×10^{8}	3.06×10^{-2}	1.00×10^{7}	2.70×10^{-4}
Tryptophan	2.0×10^{9}	3.78×10^{-2}	1.30×10^{10}	0.78	3.00×10^{8}	8.09×10^{-3}
Tyrosine	4.0×10^{8}	7.55×10^{-3}	1.30×10^{10}	0.78	2.80×10^{8}	7.55×10^{-3}
Valine	1.2×10^{7}	2.27×10^{-4}	8.50×10^{8}	5.11×10^{-2}	1.00×10^{7}	2.70×10^{-4}

4. Supplemental results

4.1 Effect of timestep

The timestep is an important parameter in the simulation.

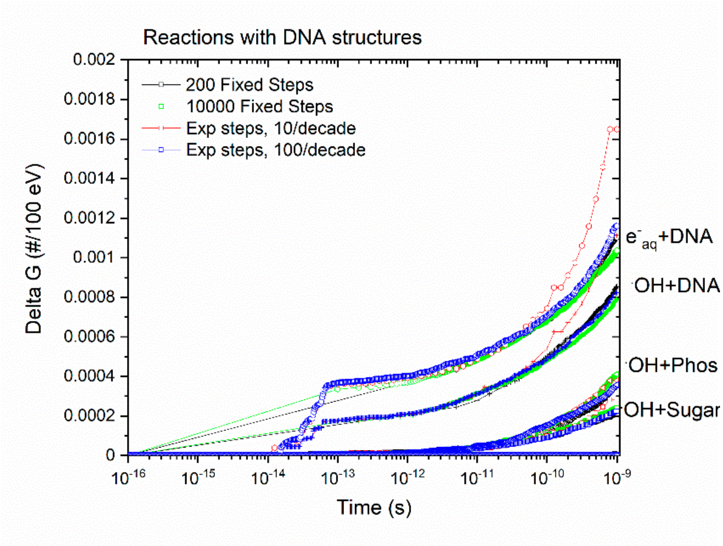

Figure S3. Delta-G for selected reactions with DNA, using fixed or exponentially growing time steps.

4.2 Z scores for the DFT peak

The DFT data is very noisy. To put the peak of the DFT at 0.1 bp^{-1} into evidence, we calculated the mean and standard deviation of the noise, excluding the first two points (the large peak at 0 bp^{-1}). Using these values, we calculated the Z scores for the peak at 0.1 bp^{-1}. Here are the Z scores calculated for the photons and ions. In general, the Z scores are higher when histones are present.

Table S30. Z scores of the peak at 0.1 bp^{-1}.

Ion	Energy	LET	Without histones, 1st strand	Without histones, 2nd strand	With histones, 1st strand	with histones, 2nd strand
Photon	100	0.3	3.09229	2.46772	3.89454	5.20341
H	100	0.73	3.5103	5.55107	5.68594	4.05668
H	50	1.24	3.12391	3.14965	4.96967	5.61719
H	20	2.61	2.57861	2.35089	4.52619	5.32565
H	10	4.59	3.46074	2.7179	5.37888	5.10974
H	5	8	1.57371	4.92607	4.62952	6.36757
H	3	11.94	4.7087	2.35258	5.8844	4.82653
H	2	16.26	3.91593	3.16786	4.87871	6.63395
H	1	26.94	2.79916	4.20137	5.87155	5.56214
He	100	2.91	3.61493	2.63869	4.52149	5.03128
He	50	4.97	1.87189	1.52906	5.31935	5.42273
He	30	7.5	2.43508	1.13952	2.78623	3.73404
He	20	10.44	3.03995	2.37034	4.54759	5.32915
He	10	18.35	1.33212	0.81907	5.05763	4.31843
He	5	31.98	4.40604	1.73284	5.86965	4.8809
He	3	47.57	4.73423	1.94395		
He	2	64.31	0.74575	0.4259	4.8857	5.80773
He	1	102.8	3.23886	1.49455	4.11783	4.81733
C	100	26.2	0.20341	2.96986	5.47911	4.97142
C	50	44.76	3.54534	1.63426	3.88953	1.95631
C	30	67.51	3.71672	3.78633	5.56367	5.74488
C	20	93.84	4.13602	3.19038	5.62052	4.23672
C	10	163.77	2.01264	3.68185	4.59681	5.0402
O	325	21.5	3.73107	3.73818	3.52206	5.59672
Fe	1000	150	2.65167	0.92061	5.42371	4.52368
